# The Effects of Digital Technology on Opportunity Recognition

**DOI:** 10.1007/s12599-021-00733-9

**Published:** 2022-02-03

**Authors:** Thomas Kreuzer, Anna-Katharina Lindenthal, Anna Maria Oberländer, Maximilian Röglinger

**Affiliations:** 1 FIM Research Center, Project Group Business and Information Systems Engineering of the Fraunhofer FIT, University of Augsburg, Universitätsstraße 12, 86159 Augsburg, Germany; 2 FIM Research Center, Project Group Business and Information Systems Engineering of the Fraunhofer FIT, University of Bayreuth, Wittelsbacherring 10, 95444 Bayreuth, Germany

**Keywords:** Opportunity recognition, Digital entrepreneurship, Digital technology, Digital technology effects, Digital innovation

## Abstract

Recognizing opportunities enabled by digital technology (DT) has become a competitive necessity in today’s digital world. However, opportunity recognition is a major challenge given the influence of DT, which not only disperses agency across various actors, but also blurs boundaries between customers, companies, products, and industries. As a result, traditional entrepreneurship knowledge needs to be rethought and the effects of DT on opportunity recognition need to be better understood. Drawing from opportunity recognition theory – as one of the central theories in the entrepreneurship domain – this study builds on a structured literature review to identify and explain three direct as well as three transitive effects of DT on opportunity recognition. These effects have been validated with real-world cases as well as interviews with academics and practitioners. In sum, this study contributes to descriptive and explanatory knowledge on the evolution from traditional to digital entrepreneurship. As a theory for explaining, the findings extend opportunity recognition theory by illuminating how and why DT influences opportunity recognition. This supports research and practice in investigating and managing opportunities more effectively.

**Supplementary Information:**

The online version contains supplementary material available at 10.1007/s12599-021-00733-9.

## Introduction

Thomas Edison presented the first light bulb in 1879. Today, digital technology (DT) influences the entrepreneurial endeavors of lighting companies by pushing the innovation limits of their light bulbs. For instance, If This Then That (IFTTT) internet services enable reprogrammable light bulbs that can be controlled via mobile applications. Recognizing the potential, Philips developed Hue bulbs which, for example, warn against burglars (ifttt.com/hue 2020). In this example, DT enabled novel opportunities that Philips leveraged in the form of the digitally connected and re-programmable Hue bulbs.

Speaking more generally, DT creates novel opportunity spaces for entrepreneurial endeavors (Ciriello et al. 2018; Henfridsson et al. 2018; Oberländer et al. 2021). It enables digitalizing functions of and adding digital capabilities to physical products (Yoo et al. 2010), and hence provides new ways of interaction between customers and companies (Lokuge et al. 2019). Despite the long-standing history of entrepreneurship, recognizing opportunities in a digital world is a major challenge, as the unique characteristics of DT (e.g., re-programmability, data homogenization, self-referential nature) differ from those of other technologies (Yoo et al. 2010). DT challenges and reshapes existing assumptions (von Briel et al. 2021), for example, by dispersing agency across various actors as well as by blurring boundaries between customers, companies, products, and industries (Oberländer et al. 2021; Yoo et al. 2010). As DT has pervasive societal and economic effects (Baskerville et al. 2020), it becomes increasingly difficult for research and practice to apply and draw from traditional entrepreneurship knowledge of opportunity recognition (Nambisan 2017; Steininger 2019). Hence, researchers have taken on the challenge to study opportunity recognition in a digital world, engaging in the comparably new research stream *digital entrepreneurship* (DE).

In DE, important findings regarding opportunity recognition originate from traditional entrepreneurship research, where scholars argue that opportunities are key to entrepreneurial endeavors (Shepherd et al. 2019). Research, for instance, applied entrepreneurship nexus theory to understand the emergence of opportunities (Davidsson 2015). In addition, authors studied opportunity recognition from a process perspective in terms of activities, input, and outcome, e.g., Ardichvili et al. (2003), and from a behavioral perspective focusing on an individual’s behavior when engaging in opportunity recognition, e.g., Baron (2007). More recently, the evolution from (traditional) entrepreneurship to DE research shifted the focus towards investigating the nature of opportunities enabled or influenced by DT (Recker and von Briel 2019). In this context, many DE studies have already focused on DT influencing opportunities as promising for future research, such as von Briel et al. (2021) or Nambisan (2017). Among the few contributions that explicitly studied digital opportunities, Oberländer et al. (2021) conceptualized the digital opportunity space for incumbents and Secundo et al. (2021) examined DT-enabled opportunities for educating entrepreneurship.

In sum, research has still not fully explored and understood the evolution from entrepreneurship to DE, although it acknowledges that there is a differentiation between both (Nambisan 2017; von Briel et al. 2021). Significantly, research lacks a profound understanding of the effects of DT on opportunity recognition (von Briel et al. 2021), one of the central theories in the entrepreneurship domain (Baron and Ensley 2006; Shepherd et al. 2019). Thus, many DE studies have called for a better understanding, e.g., Recker and von Briel (2019) by asking “how do digital technologies assist with the discovery or creation of opportunities” (p. 5). However, neither fundamental constructs of opportunity recognition nor more detailed aspects of the process and behavioral perspectives have been studied with a focus on the effects of DT (Recker and von Briel 2019; Steininger 2019). The effects of DT on opportunity recognition are of particular interest not only in the DE domain, but also in related domains such as digital innovation, where opportunity recognition – as a first step in the innovation process – is still regarded understudied, too (Abrell et al. 2016; Ciriello et al. 2018; Holmström 2018). The lack of understanding hinders scientific progress and practitioners are left without guidance on how to best recognize opportunities in a digital world (Shen et al. 2018; Svahn et al. 2017). Against this backdrop, we conclude that understanding the effects of DT on opportunity recognition is essential to advance DE research and practice (Nambisan 2017) and provides valuable insights into the evolution from entrepreneurship to DE. Thus, we ask: *What are the effects of digital technology on opportunity recognition?*

To address this research question, we draw from opportunity recognition theory – as one of the central theories in the DE domain – aiming to conceptualize the effects of DT on opportunity recognition and to explain the evolution from traditional entrepreneurship to DE. In a first step, we derive four key constructs of opportunity recognition theory from the traditional entrepreneurship literature. In a second step, we build on a structured literature review (vom Brocke et al. 2015), complemented with coding techniques for theorizing by Wolfswinkel et al. (2013) to identify the effects of DT on these key constructs. To this end, we build on the fact that DT enables a close link between opportunity recognition in DE and digital innovation (von Briel et al. 2021) which allows us to draw from mature knowledge about DT in the digital innovation literature. As a result, we identify three direct as well as three transitive effects of DT on opportunity recognition and provide rationales for each effect. Finally, we validate the effects with secondary data from real-world cases and through semi-structured expert interviews with scholars and practitioners (Myers and Newman 2007). Our work contributes to the descriptive and explanatory knowledge of the evolution from traditional entrepreneurship to DE, whereby we consider our results as a theory for explaining, which addresses the question how and why DT influences opportunity recognition (Gregor 2006). Thus, we extend opportunity recognition theory by providing a validated starting point for further theorizing on opportunity recognition in the digital context.

The remainder of this paper is structured as follows: Next, we elaborate on traditional entrepreneurship and DE as domain background, and on DT and opportunity recognition theory as theoretical background. Thereafter, we outline our research method before introducing our results, i.e., the effects of DT on opportunity recognition. We conclude by discussing limitations and stimuli for further research.

## Domain and Theoretical Background

To understand the effects of DT on opportunity recognition, we first outline the domain background of our study, i.e., traditional entrepreneurship and DE. As DT is a central concept in DE, we then elaborate on existing DT knowledge and, in a third step, introduce opportunity recognition theory as theoretical lens.

### Traditional Entrepreneurship and Digital Entrepreneurship

Entrepreneurship research focuses on actors, including their characteristics and context, in the process of creating new economic activities (Eckhardt and Shane 2003; Shepherd et al. 2019). Thereby, research studies entrepreneurial endeavors (e.g., the exploration and exploitation of opportunities), corresponding resources required (e.g., cognitive resources), as well as entrepreneurial processes and activities to achieve these endeavors (e.g., recognizing opportunities) (McMullen and Dimov 2013; Schumpeter 1934; Shepherd et al. 2019). Thereby, entrepreneurship research is particularly interested in new phenomena influencing entrepreneurial endeavors (Recker and von Briel 2019; Shen et al. 2018) such as technological change (Shane 2000). Most prominently, emerging DT is changing entrepreneurship research in various facets (Nambisan et al. 2017; von Briel et al. 2021), whereby Del Giudice and Straub (2011) describe the influence of DT as “the magic ingredient that inspires and most often enables contemporary entrepreneurial endeavors” (p.iii). In this regard, entrepreneurs using DT for entrepreneurial activities are the core of DE research (Block et al. 2020; Gustavsson and Ljungberg 2018). Related work studies, for instance, entrepreneurship from a high-level DT perspective (Nambisan 2017), digital entrepreneurial ecosystem (Sussan and Acs 2017), or the roles DT can take in entrepreneurial endeavors (von Briel et al. 2021). To complement this growing body of (macro-level) knowledge of future research directions, Sahut et al. (2021) constitute a need for more specific approaches.

Besides the evolution from entrepreneurship to DE, DT also enables a closer link between DE and digital innovation. Yoo et al. (2010), one of the fundamental studies of digital innovation research, introduced it “as the carrying out of new combinations of digital and physical components to produce novel products” (p. 725). Von Briel et al. (2021) analyzed and compared the DE and digital innovation domains and found clear overlaps, e.g., regarding focal phenomena or research foci. For instance, DE is interested in “the creation of new economic activities embodied in or enabled by digital technologies” (p. 3), whereas digital innovation deals with “the creation of new and improved products, processes, or services through digital technologies” (p. 3). Thus, both domains share an interest in DT as a central concept (Berger et al. 2019; Nambisan et al. 2017). In this regard, valuable work in DE, e.g., Recker and von Briel (2019) and Nambisan et al. (2017), consistently draws from digital innovation literature, i.e., Yoo et al. (2010), for their understanding of DT. Finally, both domains are interested in the opportunity concept, whereby DE rather looks at the nature of opportunities and entrepreneurial activities (von Briel et al. 2021). Digital innovation in contrast focuses on opportunity recognition during the initiation phase of the digital innovation process (Kohli and Melville 2019; Nambisan et al. 2017).

Due to the high importance of DT for both and a shared interest in opportunity recognition, there is a close link between DE and digital innovation research. Thus, we argue that digital innovation literature can be used to increase our understanding of DT-related phenomena in DE research, e.g., for revising existing and for developing new theories on the influence of DT (Berger et al. 2019). In terms of our study, this relationship contributes to our understanding of the evolution from entrepreneurship to DE by examining the role of DT in recognizing opportunities (Nambisan et al. 2017; von Briel et al. 2021).

### Digital Technology

As our study focuses on identifying the effects of DT on opportunity recognition, we are interested in existing conceptualizations of DT that we can potentially leverage for our literature analysis and for generating explanatory insights. Among the few studies that directly address the link between DT and entrepreneurship, von Briel et al. (2021) proposed three roles DT can take in entrepreneurial endeavors, i.e., as an enabler, outcome, or as context. Beyond this, recent contributions show that the scope and boundaries of DT have not yet been consistently defined (Baskerville et al. 2020; Faulkner and Runde 2019). Often described as the use of digital resources to extract, create, analyze, communicate, or use information in specific contexts (Zuppo 2012), DT is commonly used as an umbrella term for information technology (IT) in the context of digitalization (Denner et al. 2018). Further, there are many concepts that are similar to and not clearly differentiated from DT, e.g., digital objects (Faulkner and Runde 2019), digital artifacts (Kallinikos et al. 2013), but also IT and IS (Baskerville et al. 2020). From an overarching perspective, research has so far studied the DT concept (1) in terms of its characteristics, i.e., differentiating it from other technologies, and (2) in terms of outcomes, i.e., DT-related outcomes of entrepreneurial endeavors (henceforth: DT outcomes) (von Briel et al. 2021).

In terms of characteristics, Yoo et al. (2010) were the first to define the constituting characteristics of DT as *re-programmability* (i.e., operational logic is separated from physical embodiment), *homogenization of data* (i.e., analogue signals are converted into binary numbers)*,* and *self-referential nature* (i.e., DT is dependent on the use of DTs). Although Yoo et al. (2010) positioned their study in the digital innovation domain, most literature in the DE domain also refers to these characteristics for their understanding of DT, e.g., von Briel et al. (2021) and Nambisan (2017). Benbya et al. (2020) expanded the three characteristics by Yoo et al. (2010) to comprise seven complexity-inducing characteristics of DT, i.e., *embeddedness*, *connectedness*, *communicability*, *editability*, *identifiability* and *associability*, which so far – due to its novelty – only a few current studies build on.

Based on these unique characteristics, DT outcomes can be distinguished from traditional artifacts in terms of convergence and generativity (Ciriello et al. 2018; Yoo et al. 2012). Convergence means that separate components of DT can be easily combined to create innovation. Generativity refers to DT’s ability to produce unprompted change, i.e., DT outcomes are indefinitely expandable. Both generativity and convergence enable novel DT outcomes, whereby in particular the *layered modular architecture*, *digital platforms* and *digital ecosystems* have been intensively discussed in the literature (Ciriello et al. 2018; Yoo et al. 2012). The *layered modular architecture* of DT manifests two relevant separations between device and service (due to re-programmability) and between network and contents (due to data homogenization) being embedded into physical objects, which enhances the object’s functionalities with digital capabilities (Yoo et al. 2010). Based on the layered architecture, DT enables the modular integration of components into *digital platforms.* Digital platforms provide an extensible base to which complementary modules (e.g., third-party software) can be added (de Reuver et al. 2018). *Digital ecosystems* refer to multiple actors, e.g., organizations and customers, who interact by means of an exchange of data, information and knowledge, and through the consumption of focal value propositions in a self-organizing, scalable and DT-mediated system, e.g., on digital platforms (Sussan and Acs 2017).

Considering the presented literature, we conclude that DT is still seen as an elusive umbrella term for which literature provides insightful characteristics and an overview of relevant DT outcomes, but no unambiguous conceptualization. As the starting point for our study, we, thus, take a high-level perspective on DT and understand it as an enabler of entrepreneurial endeavors, in particular opportunity recognition (von Briel et al. 2021). We will revert to our understanding of DT in the method and results section and elaborate on how knowledge about the characteristics of DT as well as about DT outcomes informed the identification of the effects of DT on opportunity recognition.

### Opportunity Recognition Theory

Understanding the nature of the opportunity concept has been central to entrepreneurship research and hence led to a mature body of knowledge (Davidsson 2015; Nambisan 2017; Short et al. 2010). Shane and Venkataraman (2000), for instance, describe opportunity as a means “to bring into existence new goods, services, raw materials, and organizing methods that allow outputs to be sold at more than their cost of production” (p. 451). Opportunity recognition is the first step in the entrepreneurial process, while *opportunity recognition theory* is the central theory for investigating and explaining entrepreneurial endeavors (Baron and Ensley 2006; Shepherd et al. 2019; Tumasjan and Braun 2012).

**Fig. 1:**
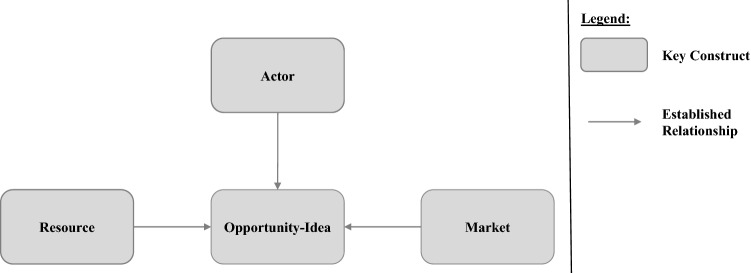
Key constructs of opportunity recognition

To date different theoretical perspectives on opportunity recognition have emerged which cover specific foci (George et al. 2016). Specifically, research studies opportunity recognition in terms of *activities*, *input*, and *outcome* from a *process perspective*, differentiates between the *discovery view* and *creation view*, and takes the *behavioral perspective* focusing on an individual’s behavior when engaging in opportunity recognition. In terms of our study, we follow Davidsson’s (2015) understanding of the opportunity concept, who argues that it should not be conceptualized as a single construct. We thus draw from the different theoretical perspectives on opportunity recognition to summarize its four key constructs shown in Fig. 1 i.e., *actor, resource, market,* and *opportunity-idea*.

Research on opportunity recognition theory chronologically followed a clear path starting in earlier work with a characterization of its fundamental constructs from a *process perspective*, e.g., Shane (2000) Eckhardt and Shane (2003). The understanding of opportunity recognition in terms of activities, input and outcome from a process perspective resembles the understanding of the digital innovation literature, which maps opportunity recognition to the initiation phase in the digital innovation process (Kohli and Melville 2019). As the starting point for the development of new processes, products, services, or business models, the process of opportunity recognition requires an *actor*, i.e., an organization and/or individual (Damanpour and Wischnevsky 2006; Davidsson 2015).

Taking the process perspective, Ardichvili et al. (2003) defined opportunity as a “chance to meet a market need (or interest or want) through a creative combination of resources to deliver superior value” (p. 108). Along these lines, they unfolded opportunity recognition into three distinct activities: *Perception*, i.e., “sensing or perceiving market needs and/or underemployed resources” (p. 109), *discovery*, i.e., “discovering a fit between particular market needs and specified resources” (p. 109), and *creation*, i.e., “creating a new fit between heretofore separate needs and resources in the form of a business” (p. 110). In terms of the input an actor can draw from, Ardichvili et al.’s (2003) understanding indicates that an actor needs to leverage external *market*-based as well as internal *resource*-based input for opportunity recognition. The constructs *resource* and *market* are reflected in a vast body of literature related to market pull and technology (i.e., resource) push (Guo et al. 2020) which is covered in the market-based view (MBV) and the resource-based view (RBV), e.g., Shepherd et al. (2019). The MBV represents an outside-in approach, as it assumes that an organization’s market conditions, e.g., competitors, determine the starting point for opportunity recognition (Zhou et al. 2005). The RBV represents an inside-out approach. It considers internal resources to be the starting point for opportunity recognition and the inability of competitors to reproduce those as a driver of competitive advantage (Barney 1991).

Besides MBV- and RBV-related research, the activities *discovery* and *creation* defined by Ardichvili et al. (2003) evolved as two more distinct views on opportunity recognition. The discovery view assumes that an opportunity exists as an objective phenomenon, like a mountain waiting to be climbed (Shane and Venkataraman 2000). In contrast, the creation view assumes that an opportunity is created rather than discovered, referring to mountain building rather than mountain climbing. Here, the opportunity’s, i.e., the mountain’s, materialization depends on the actor’s actions (Alvarez et al. 2013), e.g., piling up earth. As these views differ fundamentally in their understanding of what an *actor* actually recognizes, there is no consistent conceptualization of the outcome of opportunity recognition. In this regard, Ardichvili et al.’s (2003) rather vague view of discovering or creating a “fit” between market needs and available resources was refined in more recent research, e.g., by Shepherd et al. (2019) equating opportunity recognition with the identification of one or more ideas. Other literature also linked opportunity with the concept of an idea, i.e., as a thought or suggestion to act on an opportunity (Kornish and Ulrich 2011; Nambisan et al. 2017). Finally, Davidsson (2015) defined *new venture ideas* as the outcome of opportunity recognition and suggested the term *opportunity-idea*, which we use, as an alternative label. The *opportunity-idea* thereby combines the concept of an opportunity, as a possibility for action, with the concept of an idea (Shen et al. 2018), and we understand it to be the central outcome of opportunity recognition. On the one hand, the *opportunity-idea* may pre-exist as a source to be discovered by the *actor* (Abrell et al. 2016). On the other hand, the *opportunity-idea* can be created by the *actor* (Kohli and Melville 2019). In doing so, we follow Gustavsson and Ljungberg (2018) by taking a deliberately broad understanding of the *opportunity-idea* from a first-person perspective, i.e., it is actor-specific and hinges on an actor’s context and characteristics, that covers both the discovery and creation view.

Although research on opportunity recognition always considered it to be a process, Baron and Ensley (2006) and Baron (2007) criticized that the question of how the process of opportunity recognition occurs in the mind of an actor, i.e., the cognitive process(es), has not been sufficiently addressed so far. This motivated the *behavioral perspective* (Hulbert et al. 2015), also referred to as *cognitive perspective* (e.g., Lorenz et al. (2018) and Tumasjan and Braun (2012)), which focuses on the cognitive ability and behavior of an actor engaging in opportunity recognition. This includes research studying the role of factors such as knowledge, alertness, intuition or creativity, and behaviors favorable to opportunity recognition that trigger corresponding cognitive processes, e.g., Baron (2007) and Dyer et al. (2008). As shown by Kuckertz et al. (2017), the process and behavioral perspective are tightly linked and difficult to untangle as the activities related to the process of opportunity recognition, e.g., perception (Ardichvili et al. 2003), are mostly cognitive and hence influenced by an actor’s cognitive abilities and behavior. Consequently, entrepreneurship research has developed both perspectives simultaneously to advance knowledge on opportunity recognition, e.g., Grégoire et al. (2010). To increase the clarity of our work, we understand the process perspective to refer to *what* activities, input and outcome relate to the process of opportunity recognition, whereas we understand the behavioral perspective to refer to *how* an actor is able to carry out corresponding activities, e.g., based on its cognitive ability and behavior. Due to the strong focus of the behavioral perspective on individuals engaging in opportunity recognition, we understand this perspective to be implicitly represented in the construct *actor*.

In line with the presented literature on opportunity recognition, we also define three established relationships between the four key constructs shown in Fig. 1. The *market* shapes the *opportunity-idea*, which is grounded in the *actor’s resource* (Ardichvili et al. 2003). On the one hand, the *market* focuses on the *market* situation of the *actor* that influences the generation of an *opportunity-idea*, e.g., characterized by the *actor*’s position on the *market* and in relation to other *market* participants (Brem and Voigt 2009). On the other hand, *resource* relates to the *resource* base (e.g., assets and capabilities) available to the *actor* that shapes the generation of an *opportunity-idea*.

In sum, the literature provides extensive knowledge on opportunity recognition theory, thus underlining that it is one of the most important theories for entrepreneurship research (Baron and Ensley 2006; Dyer et al. 2008; Shepherd et al. 2019). Hence, studying it in digital contexts will provide valuable insights to increase our understanding of the evolution from traditional entrepreneurship to DE. To do so, we believe that research needs to revisit the paths taken by traditional entrepreneurship scholars to first understand what effects of DT influence opportunity recognition on a conceptual level, before deep-diving into more detailed, empirical investigations of specific effects. We argue that the four key constructs as shown in Fig. 1 sufficiently address our need for a well-founded basis for exploring the effects of DT on opportunity recognition. Thereby, we integrate mature knowledge on opportunity recognition in terms of activities, input and outcome from the process perspective, i.e., *actor, resource*, and *market,* as well as the discovery and creation view, i.e., *opportunity-idea,* and implicitly consider the behavioral perspective via the construct *actor* and corresponding relationships.

## Research Method

**Fig. 2:**
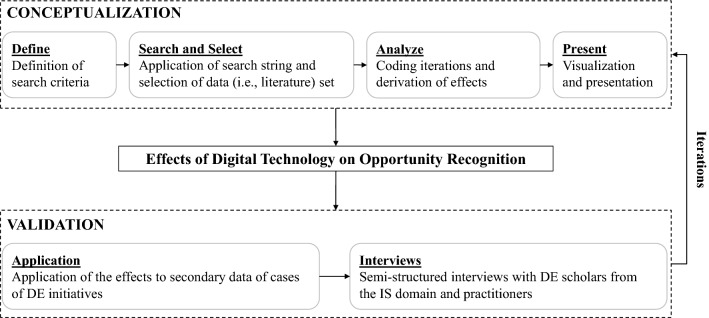
Two-stage research approach

To identify the effects of DT on opportunity recognition, we followed a two-stage research approach (Fig. 2). During the *CONCEPTUALIZATION* stage, we analyzed and synthesized literature relevant to our research question (vom Brocke et al. 2015; Wolfswinkel et al. 2013). During the *VALIDATION* stage, we followed Gregor (2006) who emphasized the need for validation against predefined criteria.

### Conceptualization

During the *CONCEPTUALIZATION* stage, we conducted a structured literature review (vom Brocke et al. 2015) complemented with coding techniques for theorizing developed by Wolfswinkel et al. (2013). This approach is particularly useful for the data-based development of a well-structured set of constructs and corresponding relationships, which in our case relates to identifying and conceptualizing effects of DT on opportunity recognition. The literature review comprised four steps (Table 1).

**Table 1:** Conceptualization of the DT effects with a structured literature review

(1) Define	(2) Search and select	(3) Analyze	(4) Present
Definition of search criteria	Application of search string and selection of data set	Coding iterations and conceptualization of the effects of DT on opportunity recognition	Visualization and presentation
*Topic* IS domain search string “digital innovation” *Quality filter* IS senior scholars’ basket ECIS and ICIS Journals (impact factor > 3)	*Topic search* n = 729 (*Web of Science* = 300; *AIS eLibrary* = 429) *Quality and duplet check* n = 154 *Relevance check* n = 53 *Forward and backward search* n = 6 *Final sample* **n = 59**	*Preparation* Extracting excerpts from the literature Sample relevant to the phenomenon of DT influencing opportunity recognition **Open Coding** Deriving 111 open codes regarding the Effects of DT on opportunity recognition from the excerpts **Axial and Selective Coding** *First iteration* Deriving 44 single selective codes that provide descriptive and explanatory insights Regarding the effects of DT on opportunity recognition Identifying six effects of DT on the key constructs of opportunity recognition *Second iteration* Identifying and substantiating three digital phenomena that characterize the enabling role of DT driving the effects Deriving a set of 15 rationales that explain how the digital phenomena enable the effects of DT	*Presentation* Illustrating the effects of DT on the key constructs of opportunity recognition

The step *define* aims at developing a set of search criteria to identify relevant publications. Our research topic is rooted in the IS domain and our research question requires a data set of high-quality research. Hence, we focused our search on high-impact IS journals and conference publications, i.e., the IS Senior Scholars’ Basket of Journals (AIS 2011), the International Conference on Information Systems (ICIS), and the European Conference on Information Systems (ECIS) (Bandara et al. 2015). In addition, we included publications from other journals with an impact factor of more than three. Considering our research question, we are particularly interested in how DT influences opportunity recognition. The DE literature has so far paid little attention to understanding the characteristics and effects of DT and, instead, draws from literature in related domains, e.g., digital innovation (Yoo et al. 2010), for its understanding of DT. To this end, we build on the fact that DT enables a close link between opportunity recognition in DE and digital innovation (von Briel et al. 2021), allowing us to draw from knowledge on the umbrella term DT in the digital innovation literature. Against this backdrop and to be in line with other literature reviews, e.g., Kohli and Melville (2019), we kept our search term broad and simple and defined “digital innovation” to appear within the topic field specified by the Web of Science search engine. Besides Web of Science, we used the AIS eLibrary to identify relevant conference publications. During the step *search and select*, we conducted a rating to identify relevant publications and ended with a final set of 59 publications to analyze. In Online Appendix 1 (available online via http://link.springer.com), we provide an overview of what is within and outside the scope of our study (Cram et al. 2016), and detailed information on the rating process.

After compiling the data set, we aimed at deriving the effects of DT on opportunity recognition during the step *analyze* by using the coding techniques for theorizing of Wolfswinkel et al. (2013). We started by reading each publication in our data set and highlighted relevant findings and insights regarding DT influencing opportunity recognition, i.e., excerpts. To decide on relevant excerpts, we focused on insights regarding the four key constructs representing opportunity recognition theory as a theoretical lens, i.e., *actor, resource, market,* and *opportunity-idea* (see Fig. 1). Regarding DT, the literature does not provide an unambiguous conceptualization of the umbrella term DT (see digital technology section) and papers in our literature sample rely on different, partly inconsistent conceptualizations (see also Table A6 for an overview of which conceptualization of DT is used by the publications in our data set). Hence, we decided to extract excerpts that broadly address one of the four key constructs in digital contexts or the enabling role of DT (von Briel et al. 2021) for opportunity recognition. Thereafter, we aimed to decide on an appropriate conceptualization of DT for further DT-related analysis and sensemaking during the coding process.

According to Wolfswinkel et al. (2013), researchers should engage in three coding iterations during and after extracting excerpts: open, axial, and selective coding. During open coding, we re-read all excerpts and derived a set of 111 open codes, i.e., individual terms, phrases and sentences, that “capture parts of the excerpted data set” (Wolfswinkel et al. 2013: 51). Considering that we already extracted the excerpts referring to the four key constructs of opportunity recognition, we were able to map each of the open codes to at least one key construct. For the implementation, one author coded and mapped and another checked and confirmed/edited the results before the whole author team discussed and refined them. We conducted axial and selective coding in two iterations: During the first iteration of axial coding, one author initially identified interrelations between codes, this was checked by a second author and then again discussed, developed, and adapted by the whole author team. We combined and clustered these interrelated codes in terms of higher levels of abstraction (Wolfswinkel et al. 2013). The first iteration of the axial coding revealed two kinds of insights. One the one hand, descriptive insights revealed how the constructs of opportunity recognition have been affected given the influence of DT. We found two types of effects of DT: One effect type directly influences the *actor*, *resource* and *market* constructs while another effect type transitively influences the *opportunity-idea* through one of the other constructs. On the other hand, we gained explanatory insights into the enabling role of DT by addressing how and why DT influences the constructs. During the first iteration of the selective coding, we further refined the results of the axial coding and ended with 44 single selective codes which we used to derive six effects of DT on the key constructs of opportunity recognition (see Table A6 and A7 for an overview of which publications in our data set revealed which selective codes).

To enhance and substantiate the explanatory insights regarding the six effects of DT on opportunity recognition, we conducted a second iteration of axial and selective coding. At first, we attempted to make further sense of the six effects of DT by using prominent characterizations of DT, e.g., Yoo et al. (2010) and Benbya et al. (2020), to structure the results of the first iterations, i.e., effects and selective codes. However, we realized that the characteristics of DT are inconsistently used in literature, are closely interrelated in terms of impact, making it impossible to relate individual characteristics to effects, and that studies mostly understand the DT concept as a general umbrella term (Baskerville et al. 2020; Denner et al. 2018). Instead, we found that DT affects opportunity recognition as a holistic enabler and that the effects of DT are driven by three digital phenomena which build on DT outcomes through which the digital phenomena (mainly) emerged (see theoretical background section). Following Wolfswinkel et al.’s (2013) suggestion to combine inductive and deductive thinking for axial and selective coding, we also drew from renowned IS literature as justificatory knowledge to increase our understanding of the DT-related phenomena. Doing so, and by continuously re-reading the justificatory references, excerpts, and codes from our data set, we developed rationales that disclose how and why the digital phenomena and DT outcomes drive the effects of DT on opportunity recognition. During the second iteration, we conducted axial and selective coding until we reached theoretical saturation (see Appendix 1 for an overview of the final data set of 59 references and the effects of DT they revealed). In line with Wolfswinkel et al. (2013), we defined theoretical saturation to be achieved when no new effects, digital phenomena and DT outcomes, rationales or other insights regarding our research question emerged. To ensure transparent documentation, we used the software MAXQDA for all coding efforts (Bandara et al. 2015).

As for the step *present*, we visualized our results covering the key constructs of opportunity recognition and conceptualizing the effects of DT, including digital phenomena, DT outcomes and rationales.

### Validation

During the *VALIDATION* stage, we aimed at validating the *real-world fidelity, completeness, internal consistency,* and *level of detail* of the effects of DT on opportunity recognition (Sonnenberg and vom Brocke 2012). To do so, we followed two steps: (1) Application of the effects to secondary data of real-world cases of DE initiatives, and (2) semi-structured expert interviews.

First, we applied the effects to secondary data of 34 real-world cases of DE initiatives and thereby gained initial insights regarding their *real-world fidelity* and *completeness*. For each case, we identified the involved DT and screened the data to extract why and how DT initially influenced opportunity recognition. We then assessed whether one of our effects could be identified and explained (*real-world fidelity*), and whether there were any effects missing (*completeness*). Accordingly, we assigned each case to one or more effects where appropriate. In Online Appendix 2 we provide further details regarding the data collection process, the cases, and our coding.

Second, we conducted semi-structured expert interviews (Myers and Newman 2007), which are particular useful for validating what is known but also for gaining new insights (Recker 2013). We selected seven scholars researching in DE and DT-related domains and seven practitioners working in digital contexts, based on a purposive sampling approach (Miles and Huberman 2009). During the interviews, we presented the – at that time – latest version of the effects of DT on opportunity recognition. After clarifying questions of the interviewee, we discussed the effects in general and regarding the four criteria. After each interview, the expert’s feedback was reflected in the author-team and resulting changes discussed and potentially integrated. Find further details in Online Appendix 3.

Overall, both validation steps provided valuable insights and contributed improvements to our results. Considering both validation steps, we conclude that the presented version of the effects is valid regarding the four predefined criteria and report on insights regarding practical value in the discussion section.

## Results

### Foundations: Digital Phenomena and Digital Technology Outcomes

Before we present the results, i.e., the direct and transitive effects of DT on opportunity recognition, we introduce the three digital phenomena that characterize the enabling role of DT as a foundation. As outlined in the method section, we found during our literature review that DT affects opportunity recognition in the form of a holistic enabler, whereas its characteristics are inconsistently used in literature and can neither be unambiguously differentiated nor mapped with regard to the single effects. Rather, we found that three digital phenomena, i.e., *digital invasiveness, dissolving product and industry boundaries*, and *dissolving company and customer boundaries* characterize the enabling role of DT and drive the effects of DT on opportunity recognition. These phenomena have already been acknowledged and described in the IS literature, which is why we draw from and cite related justificatory knowledge. Further, we found these digital phenomena to build on specific DT outcomes, i.e., *layered modular architecture*, *digital platforms*, and *digital ecosystems*. These DT outcomes incorporate the characteristics of DT, e.g., as outlined by Yoo et al. (2010). They are, while not necessarily being exhaustive, the most influential ones in terms of the digital phenomena based on our analysis and confirmed by justificatory knowledge.

First, *digital invasiveness* refers to DT not only changing the core of entrepreneurial endeavors but also transforming individuals’ work and personal lives (Baskerville et al. 2020; von Briel et al. 2021). This development is mainly rooted in the *layered modular architectur*e of DT, which combines the characteristics of DT according to Yoo et al. (2010), i.e., (re-) programmability, data homogenization, and its self-referential nature. The layered modular architecture enables DT to be an integral part embedded not only in digital, but also increasingly in physical everyday products invading our everyday lives. As a result, DT is omnipresent in almost everything that individuals and organizations do, thus creating a techno-society in which DT is an essential mediator of reality. Baskerville et al. (2020) used the term ‘ontological reversal’ to describe this new logic, whereby no longer represents reality but rather shapes the reality of an *actor* engaging in opportunity recognition.

Second, we found opportunity recognition to be affected by dissolving company and customer boundaries, a circumstance extending the nature and type of *resources* at the disposal of *actors*. While the RBV (Barney 1991) originally focused on the competitive advantage afforded by company-owned or -controlled resources, DT not only extends an organization’s relevant resources towards shared resources of professional partners, but also dissolves boundaries between companies and their customers. Specifically, Oberländer et al. (2021) describe how connected products (through Internet-of-Things *platforms*) in the hands of customers as well as customers’ assets and capabilities (through community-based *digital platforms*) at the disposal of incumbents close the gap between companies and their customers as they enable unprecedented proximity (Siggelkow and Terwiesch 2019). As a result, they argue for an explicit consideration of customers and their resources as shared and external resources integrated into corporate value creation and thus transcending company boundaries through *digital platforms* (Zhang et al. 2020).

Third, *digital ecosystems* dissolve product and industry boundaries (Yoo et al. 2010) in areas in which the entrepreneurial endeavors of *market* participants more than ever build on, relate to, and interfere with each other (Sahut et al. 2021), e.g., in multi-sided markets. Within digital ecosystems, the unique characteristics of DT enable digital data from heterogeneous sources, e.g., other market participants’ digital products and services, to be easily accessed, stored, transmitted, processed and (re-) combined (Baskerville et al. 2020; Yoo et al. 2010). Thus, transaction costs and market entry barriers decrease and thus make it possible for organizations to more easily enter new product-market domains beyond their current industry context (Fichman et al. 2014). As a result, DT challenges traditional assumptions of the MBV of opportunity recognition, as it increases the already existing uncertainty about and unpredictability of market conditions, but also expands market-related opportunities awaiting exploitation and exploration (Nambisan 2017).

### The Effects of Digital Technology on Opportunity Recognition

**Fig. 3:**
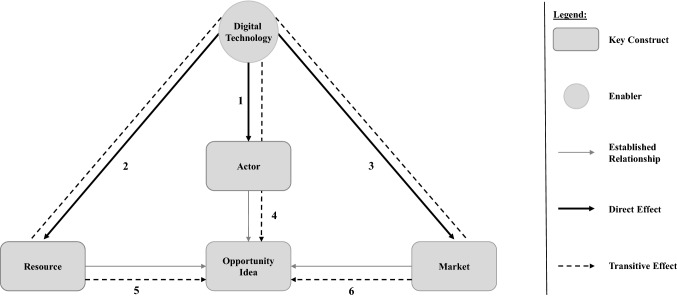
The effects of digital technology on opportunity recognition

We here present three direct as well as three transitive effects of DT on opportunity recognition (Fig. 3) as well as the digital phenomena and DT outcomes driving them. We also provide explanatory rationales for each effect, (Table 2) including references to real-world cases (Table 3).

**Table 2:** Direct and transitive effects of digital technology on opportunity recognition

#	From	To	Driven by	Through	Rationales	References
*Direct effects*						
1	Homogenous entrepreneurs	Growing number and variety of actors (everyone)	Increasing digital invasiveness	Layered modular architecture	1.1 Due to the layered architecture of DT individuals and organizations are constantly working with and surrounded by DT driving digital invasiveness and fostering digital opportunities	Ciriello et al. (2017) and Iivari et al. (2016)
					1.2 Increasing digital invasiveness fosters the ability of organizations and individuals to participate in opportunity recognition	Nambisan et al. (2017) and Yoo et al. (2012)
					1.3 DT can take a supporting or leading role as an actor contributing to opportunity recognition	Barrett et al. (2015) and Henfridsson et al. (2018)
2	Exclusively internal access only	Externally shared access	Dissolving company and customer boundaries	Digital platforms	2.1 Digital platforms enable shared access to an enlarged resource base – beyond company-owned resources – dissolving company and customer boundaries and leading to digital opportunities	Lokuge et al. (2019) and Saldanha et al. (2017)
					2.2 Digital platforms provide new digital capabilities, which digitally enhance existing products and can be shared between companies and customers for opportunity recognition	Gustavsson and Ljungberg (2018) and Yoo et al. (2012)
					2.3 Digital platforms provide new digital assets such as digital infrastructure, digital applications, and data assets, which serve as foundation for opportunity recognition	Fichman et al. (2014) and Henfridsson et al. (2018)
3	Hierarchical relationships	Multi-lateral value networks	Dissolving product and industry boundaries	Digital ecosystems	3.1 Digital ecosystems transform competitors in hierarchy-based value systems into partners for opportunity recognition by dissolving product and industry boundaries	Ciriello et al. (2018) and Oppong-Tawiah and Bassellier (2017)
					3.2 Digital ecosystems enable suppliers to contribute valuable knowledge to opportunity recognition and change existing supplier relationships	Lee and Berente (2012) and Oborn et al. (2019)
					3.3 Within digital ecosystems, regulators facilitate opportunity recognition across industries by changing legislation that explicitly targets DT	Hinings et al. (2018) and Suseno et al. (2018)
*Transitive effects*						
4	Context-dependent restrictions	Multitude of (re-) combination possibilities	Increasing digital invasiveness	Layered modular architecture	4.1 The layered architecture of DT shared by actors who are constantly surrounded by DT creates a variety of different compatible resources for opportunity recognition due to protocols and standards	Barrett et al. (2015) and Lusch and Nambisan (2015)
					4.2 The layered architecture of DT allows actors new ways of recombination for opportunity recognition through loose coupling via standardized interfaces	Henfridsson et al. (2018) and Yoo et al. (2010)
5	Deterministic and final deployment	Continuous iterative development	Dissolving company and customer boundaries	Digital platforms	5.1 By dissolving company and customer boundaries between actors, digital platforms foster the continuous adaptation and iterative refinement of ever-evolving digital artifacts	Ciriello and Richter (2015) and Gustavsson and Ljungberg (2018)
					5.2 Digital platforms enable actors to build on their own or other company’s digital artifacts as a starting point for opportunity recognition	Oborn et al. (2019) and Zapadka (2020)
6	Few occasion-related interactions	Continuous integration	Dissolving product and industry boundaries	Digital ecosystems	6.1 By dissolving product and industry boundaries, digital ecosystems enable the continuous exchange of information and sharing of knowledge between actors	Lusch and Nambisan (2015) and Suseno et al. (2018)
					6.2 Within digital ecosystems actors can communicate their needs more quickly through short product cycles, which leads to continuous opportunity recognition	Abrell et al. (2016) and Dery et al. (2017)

**Table 3:** Overview of real-world cases illustrating the effects of digital technology on opportunity recognition

#	From	To	Driven by	Through	Rationales	References
*Direct effects*						
1	Homogenous entrepreneurs	Growing number and variety of actors (everyone)	Increasing digital invasiveness	Layered modular architecture	*Danske Bank* launched digital initiatives that enhanced their employees’ digital literacy and enabled them to contribute to the DE process	Urbach and Röglinger (2019) Case 6
					*Presbyterian Church of Ghana Trinity Congregation* created an online community that allowed various church members to share their ideas and participate in opportunity recognition	Urbach and Röglinger (2019) Case 10
					*US Federal Communications Commission* used a cloud-based open innovation approach to empower employees to share solutions and take action targeted to improve their legacy systems	Urbach and Röglinger (2019) Case 21
2	Exclusively internal access only	Externally shared access	Dissolving company and customer boundaries	Digital platforms	*Airbus *leveraged external knowledge of Local Motors as a key partner to develop a crowdworking platform	Urbach and Röglinger (2019) Case 7
					*Fundación Cardiovascular (FCV)* leveraged digital platforms to develop telemedicine innovation	Urbach and Röglinger (2019) Case 8
					*Volkswagen *leveraged the external digital capabilities of startups for opportunity recognition by dissolving company boundaries	Urbach and Röglinger (2019) Case 2
3	Hierarchical relationships	Multi-lateral value networks	Dissolving product and industry boundaries	Digital ecosystems	*GKN* developed new partnerships with start-ups and involved their customers in opportunity recognition processes	Mrass et al. (2021)
					*Helix Nebula *partnered with former competitors to build a digital ecosystem for exploring new opportunity-ideas	Nambisan et al. (2020)
					*Lufthansa’s *multi-cloud architecture enabled new relationships between different stakeholders such as software companies in its digital ecosystem enabling opportunity recognition by dissolving industry boundaries	Urbach and Röglinger (2019) Case 19
*Transitive effects*						
4	Context-dependent restrictions	Multitude of (re-) combination possibilities	Increasing digital invasiveness	Layered modular architecture	*Engel* leveraged technology standards of SAP to roll-out digital process innovation across the organization	Urbach and Röglinger (2019) Case 14
					*M-Pesa *provided mobile payment services in Kenya including a micro-credit service enabled by the layered architecture of digital technology and respective standards	Nambisan et al. (2020)
					*Super Hospital Aarhus Denmark* combined “Automated Transport Service" whit a “Trolley Service” enabled by digital standards to enhance efficiency	Urbach and Röglinger (2019) Case 15
5	Deterministic and final deployment	Continuous iterative development	Dissolving company and customer boundaries	Digital platforms	*Deakin University* continuously improves its Cognitive Automation Application by integrating students’ feedback	Mocker and Fonstad (2017)
					*Go Get Car Share* analyses user data and feedback to leverage the generativity of DT to iteratively refine the platform	Hansen and Kien (2015)
					*Lego* focused on rapid prototyping based on the continuous insights of their digital platform and monitoring of their customers’ experience and a digital leadership approach	Urbach and Röglinger (2019) Case 5
6	Few occasion-related interactions	Continuous integration	Dissolving product and industry boundaries	Digital ecosystems	*Audi *‘s big data approach involved leveraging data from continuous knowledge integration implying shorter product cycles	Lacity et al. (2018)
					*Hummel’s *omnichannel retailing created continuous integration, which led to crowdsourced opportunity-ideas	Tan et al. (2017)
					*Kaeser Compressors’s* “pay-per-use” business model leveraged the digital integration to its machines to infer relevant usage information to create novel payment-related opportunity-ideas	El Sawy et al. (2016)

Overarching, we found that *digital technology* – as an enabler of entrepreneurial endeavors (von Briel et al. 2021) – influences all constructs of opportunity recognition theory. The resulting conceptualization of the effects of DT on opportunity recognition builds upon the four key constructs of opportunity recognition (domain and theoretical background section). To generate the digitally enabled *opportunity-idea*, opportunity recognition requires an *actor* (Davidsson 2015). We understand *digital technology* as the decisive construct that enables *resource*- as well as a *market*-related recognition of opportunities in digital contexts (Kohli and Melville 2019). The *opportunity-idea* is shaped by the *actor*’s *resource* base, which is enlarged by new digitally extended resources. The *opportunity-idea* is generated by an *actor* who is situated in a specific *market* environment that is expanded through digitally enabled relationships.

We conceptualize three direct effects (#1 to #3) of *digital technology* which directly influence the constructs *actor*, *resource* and *market* as well as three transitive effects (#4 to #6) of *digital technology* transitively influencing the generation of the *opportunity-idea* by changing established relationships. In doing so, we argue that there is not a single direct effect of *digital technology* on the *opportunity-idea*. Rather, the *opportunity-idea* – as the central construct of opportunity recognition – is transitively affected by *digital technology* through all other constructs. Further, all effects are moderated by the *actor*. Following the idea of causal explanations by Gregor (2006), we describe and explain the direct and transitive effects of *digital technology* based on five elements (Table 2): First, we characterize the construct or established relationship without the presence of *digital technology* (From). Second*,* we characterize the constructs as they are affected by *digital technology* (To). Third, we name the digital phenomena which we found to drive the effect (Driven by) and, fourth, the DT outcome through which the digital phenomena predominantly emerged (Through). Fifth, we list rationales that provide explanatory insights into how the digital phenomena drive the effects. We present all results in the final versions including the validation’s feedback.

#### Direct Effect #1: From Homogenous Entrepreneurs | To a Growing Number and Variety of Actors (Everyone) | Driven by Increasing Digital Invasiveness | Through Layered Modular Architecture

While in traditional entrepreneurship and innovation research the concept of *actor* used to refer to a group of mostly homogenous entrepreneurs (e.g., Schumpeter 1934), today a growing number and variety of actors can discover and create an opportunity-idea (Ciriello and Richter 2015) as well as participate in opportunity recognition in new ways, e.g., through crowdsourcing, peer production, or cultures of participation. This is mainly due to the layered modular architecture of DT (Yoo et al. 2010), enabling DT to constantly surround our business and private lives while fostering an increasing digital invasiveness (#1.1) (Baskerville et al. 2020). Organizations can leverage digital invasiveness and support opportunity recognition in digital contexts through providing correspondingly stimulating environments (Ciriello and Richter 2015; Hildebrandt et al. 2015), e.g., digital labs. Therein, actors can engage in entrepreneurial activities such as making sense of, crafting, or discussing opportunity-ideas. The *Presbyterian Church of Ghana Trinity Congregation*, for instance, established an online community, in which people propose opportunity-ideas to increase participation in worship services (Asiedu and Boateng 2019). Further, digital tools build on the layered modular architecture of DT to support the actor’s ability to recognize opportunities (#1.2). On the one hand, DT provides new communication and computing capabilities enabling individual actors to discover and create opportunity-ideas (e.g., leveraging existing digital artifacts) (Gustavsson and Ljungberg 2018; Nambisan et al. 2017). On the other hand, actors need new capabilities (e.g., new forms of creativity) to recognize opportunities due to an increasing number of recombination possibilities of digital artifacts (Ciriello and Richter 2015; Yoo et al. 2012). The case of the *Danske Bank* demonstrates how organizations can enhance their employees’ capabilities to recognize opportunities, e.g., adopting agile principles which here led to a digital payment platform (Staykova and Damsgaard 2019). Finally, DT generates a new type of non-human actors, i.e., software agents such as robots, scripts, or algorithms, that act on behalf of humans in a partly or fully automated manner or support them in opportunity recognition (#1.3), e.g., *Amazon Alexa* (Henfridsson et al. 2018).

#### Direct Effect #2: From Exclusively Internal Access only | To Externally Shared Access | Driven by Dissolving Company and Customer Boundaries | Through Digital Platforms

While traditionally a *resource* is understood to be owned and controlled by the respective actor, digital platforms allow access to externally shared assets and capabilities from multiple actors including customers, expanding the accessibility of *resources* in the digital context (Selander et al. 2013). As a result, dissolving company and customer boundaries enable actors to draw from a broader range of shared and external resources of professional partners as well as of customers when engaging in opportunity recognition (#3.1) (Arvidsson and Mønsted 2018; Lokuge et al. 2019). For instance, actors interact and collaborate on digital platforms where the convergence and generativity of DT drives (re-) combining of or loose coupling between digital artifacts (Ciriello et al. 2018; Stummer et al. 2018; Yoo et al. 2012). *Volkswagen*, for instance, connected employees via a digital lab with external (e.g., startups) and internal (e.g., employees) providers of knowledge which led to the opportunity-idea of augmented reality based virtual robot training for factories (Wildgrube et al. 2019). Digital platforms also provide new digital capabilities (e.g., novel features), which actors can embed complementarily in products and services and thereby share with other actors (#3.2) (Gustavsson and Ljungberg 2018). *Amazon Web Service*, for instance, provides a variety of digital capabilities, i.e., digital infrastructures, data analytics, and machine learning services that actors can leverage when engaging in opportunity recognition. Further, digital capabilities can be easily extended or enhanced on digital platforms by the original provider or even by other actors entitled to access, e.g., by adding new services (Gustavsson and Ljungberg 2018). Finally, new digital assets emerge, such as digital infrastructure, digital applications, and ‘data assets’ (#3.3) (Fichman et al. 2014; Henfridsson et al. 2018). For instance, actors can gain novel insights regarding their customers from data assets such as business intelligence (Fichman et al. 2014; Nambisan et al. 2019). Following this approach, *LEGO* leveraged data from customer-centric micromarketing to generate novel opportunity-ideas (e.g., *Chima* and *Ninjago*) (El Sawy et al. 2016).

#### Direct Effect #3: From Hierarchical Relationships | To Multi-lateral Value Networks | Driven by Dissolving Product and Industry Boundaries | Through Digital Ecosystems

Traditionally, the *market* as an organizational form of economic activities covers hierarchical relationships coordinating selected market participants, which limits the ability of organizations to recognize opportunities (Abrell et al. 2016; Berkemeier et al. 2019). DT disrupts this traditional logic as digital ecosystems dissolve product and industry boundaries (Ciriello et al. 2018; Yoo et al. 2010). Within digital ecosystems, actors establish multi-lateral value networks, where former competitors may become partners for opportunity recognition (#2.1) (Nischak and Hanelt 2019; Törmer 2018). An actor can engage in value-adding partnerships with new and/or existing other market participants across product and industry boundaries by sharing and jointly developing opportunity-ideas (e.g., joint software development with competitors) (Ciriello et al. 2018). This is demonstrated by real-world cases like *GKN*, a manufacturer of high-precision parts for the automotive industry that formed a new partnership with a 3D printing startup. Together they implemented a new business model and *GKN* recognized the opportunity-idea for bringing metal additive manufacturing technology to their customers to manufacture precision components (Wildhirt et al. 2019). Digital ecosystems also allow actors to expand collaboration efforts with their suppliers contributing knowledge to opportunity recognition (#2.2) (Fichman et al. 2014; Oborn et al. 2019). *Lufthansa*, for instance, developed the prototype of a flight scheduling application together with one of its cloud providers after intensifying their knowledge exchange regarding the potentials of digital architectures (Somoskői et al. 2019). At last, regulators may change the market by creating DT-related legislation (#2.3) (Hinings et al. 2018; Suseno et al. 2018). For instance, the COVID-19 pandemic has demonstrated how legislation, i.e., restriction of physical interactions, can affect opportunity recognition, i.e., organizations being forced to digitalize their business.

#### Transitive Effect #4: From Context-Dependent Restrictions | To a Multitude of (re-) Combination Possibilities | Driven by Increasing Digital Invasiveness | Through Layered Modular Architecture

Traditionally, non-standardized and tightly coupled components of artifacts, which cannot be decomposed or re-combined, led to context-dependent restrictions limiting the recognition of opportunities (Ulrich 1995). DT extends or even removes those boundaries given its layered modular architecture (Yoo et al. 2010). These layers lead to DT being omnipresent for a growing number of actors which are given more and more (re-) combination possibilities for opportunity-ideas. First, this is enabled due to protocols and standards increasing the number of compatible resources for actors (#4.1) (Barrett et al. 2015; Yoo et al. 2010). *Super hospital Aarhus*, for instance, combined existing services, sensors, and mobile devices based on DT standards (i.e., IHE and HL7) to develop a tool that automates the generation of tasks and notifications (Meister et al. 2019). Second, standardized interfaces (e.g., APIs) increase (re-) combination possibilities by enabling loose coupling of different DT layers (#4.2) (Henfridsson et al. 2018; Yoo et al. 2012). Actors can leverage the layered modular architecture of DT for use as well as design recombination. Use recombination describes actors connecting digital resources that are currently in use to create an individual value, while design recombination describes actors connecting digital resources as a value to users (Henfridsson et al. 2018). *M-Pesa*, for instance, leveraged design recombination by offering innovative mobile payment services in Kenya. Due to standardized interfaces, *M-Pesa* was able to couple already existing devices with a new micro-credit service. Kenya’s population might adopt the offered services and further combine it with other services (e.g., electronic wallets) as use recombination (Markus and Nan 2020).

#### Transitive Effect #5: From Deterministic and Final Deployment | To Continuous Iterative Initiation | Driven by Dissolving Company and Customer Boundaries | Through Digital Platforms

Traditionally, deterministic and final deployment of artifacts hampered subsequent changes and improvements of products and services (e.g., Lokuge et al. 2019). Today’s actors can leverage digital platforms dissolving company and customer boundaries by continuously adapting and iteratively refining ever-evolving digital artifacts (#6.1). Opportunity-ideas can be continuously edited and enhanced due the malleability of DT, i.e., generativity, leading to continuous deployment and refinement (Huang et al. 2017). For instance, *Go Get Car Share* provides a digital platform, on which actors can share their car. After the release of early versions of new features or services, i.e., minimum viable products, *Go Get Car Share* analyzes user data and leverages the re-programmability of DT to iteratively refine the platform (Tan et al. 2017). Thereby, DT provides almost endless flexibility for actors to create and discover opportunity-ideas that include the modification of existing digital artifacts (#6.2). In terms of programmable digital artifacts, an actor can pick up on existing solutions after the initial design and deployment leading to continuous refinement of opportunity-ideas (Oborn et al. 2019). *LEGO* facilitates the platform-based development of products where partners can pick up or further develop existing or already implemented opportunity-ideas (i.e., products and services) (El Sawy et al. 2016).

#### Transitive Effect #6: From Occasion Related Interactions | To Continuous Integration | Driven by Dissolving Product and Industry Boundaries | Through Digital Ecosystems

While actors were traditionally limited by their products to occasion related interactions, e.g., with their customers at the point of sales (e.g., Saldanha et al. 2017), digital ecosystems enable continuous interactions and the continuous integration of actors for the generation of opportunity-ideas. This is due to the ecosystem-driven dissolving of product and industry boundaries through which multiple actors (e.g., customers, employees, stakeholders) can easily engage with each other to continuously share information and knowledge (#5.1) (Dery et al. 2017; Lusch and Nambisan 2015). Actors can leverage digital ecosystems to recognize new opportunity-ideas in their market environment (Lusch and Nambisan 2015), especially with knowledge about customers and knowledge obtained from customers (Abrell et al. 2016; Suseno et al. 2018). For instance, *Audi* gained insights from analyzing vast amounts of data from their digital customer channels and was hence able to improve the effectiveness of their sales processes (Dremel et al. 2017). In turn, participants in digital ecosystems can also actively communicate their needs more quickly which leads to rapid adaptation to those needs and shorter development cycles (#5.2) (Abrell et al. 2016). Actors increasingly build products and services around the participation of and communication with other market participants (e.g., crowdsourcing, collaborative sharing economy, on-demand online services) (Suseno et al. 2018). For instance, *Hummel’s* leveraged its omnichannel strategy to create continuous customer integration (e.g., research and shop anywhere) and identified opportunity ideas by screening their social media platforms (Hansen and Kien 2015).

## Discussion

### Contribution

Although the opportunity concept and opportunity recognition theory are at the core of the (digital) entrepreneurship domain (Baron and Ensley 2006; Shepherd et al. 2019; Short et al. 2010), the DE literature has not yet comprehensively addressed essential questions regarding digital opportunities (Oberländer et al. 2021; von Briel et al. 2021). These open questions specifically relate to DT enabling the evolution from entrepreneurship to DE (Block et al. 2020), as it challenges existing assumptions and requires theories to be re-examined (Berger et al. 2019). To date, research specifically lacks a profound understanding of the effects of DT on opportunity recognition (Steininger 2019; von Briel et al. 2021). This is why DE scholars advocate a need “to incorporate digital technology into their theorizing” (von Briel et al. 2021: 16), specifically calling for expanding scientific knowledge on how DT influences opportunity recognition (Nambisan 2017; Steininger 2019). We followed this call by asking *what are the effects of digital technology on opportunity recognition.*

To address this question, we draw from existing knowledge on opportunity recognition theory as theoretical lens – and as one of the central theories in the entrepreneurship domain – that guides our understanding and the identification of the key constructs related to opportunity recognition. Building on a structured literature review (vom Brocke et al. 2015), complemented with coding techniques for theorizing by Wolfswinkel et al. (2013), we make a twofold contribution: First, we identified three direct and three transitive effects of DT on opportunity recognition. Regarding the specific role of DT, we found that specific characteristics of DT, e.g., as proposed by Yoo et al. (2010), are inconsistently used in literature, that they are closely interrelated in terms of impact, making it impossible to relate individual characteristics to effects, and that studies mostly understand the DT concept as a general umbrella term (Baskerville et al. 2020; Denner et al. 2018). Instead, we consider DT as a general enabler whose characteristics jointly lead to digital phenomena, i.e., *digital invasiveness*, *dissolving product and industry boundaries*, and *dissolving company and customer boundaries*. These phenomena emerged during our literature analysis and were confirmed by justificatory knowledge. Second, we relate each effect to an underlying digital phenomenon driving it that builds on DT outcomes through which the digital phenomena predominantly emerged. Finally, we provide rationales that explain how and why the effects occur.

In sum, our work complements existing DE research and contributes to the descriptive and explanatory knowledge of opportunity recognition in the digital context (Leidner 2018; Seidel and Watson 2020). Leidner (2018) states that, before building explanations, a summarization and analysis of prior knowledge is needed, which in our case refers to the findings of the structured literature review. Further, Seidel and Watson (2020) define explanations to “create understanding often through specifying causal mechanisms or processes” (p. 288), which corresponds to the digital phenomena, DT outcomes, and rationales we provide. Hence, we regard our work as a theory for explaining, i.e., a type II theory in terms of Gregor (2006), by addressing how and why DT influences opportunity recognition (Leidner 2018). Further, as the effects reveal how the constructs of opportunity recognition theory evolved given the influence of DT, they also provide valuable insights into the evolution of traditional entrepreneurship to DE.

### Theoretical Implications

Our work connects to the ongoing discussion on the effects of DT on entrepreneurial endeavors in DE research (Berger et al. 2019). In this regard, our theoretical implications are threefold, providing a starting point for further theory and method development in opportunity recognition research, a basis from which to study the process and behavioral perspective of opportunity recognition in digital contexts, and insights into the opportunity concept in relation to the *resource* and *market* constructs.

First, our findings represent a fundamental step towards sound scientific methods for theory development and validation regarding opportunity recognition in the digital context, e.g., toward theories for predicting (i.e., Types III–IV) as well as design and action (i.e., Type V) (Gregor 2006). In terms of predictions, future research can build on the identified effects and conduct quantitative empirical studies to substantiate but also expand the provided explanations towards predictive knowledge. In this regard, it may be particularly interesting, for example, to investigate a potential link between the effects of DT and the success of subsequent DE initiatives. In terms of design and action, research and particularly practice would benefit from a replicable method for generating opportunities in digital contexts. The descriptive and explanatory knowledge we provide can serve as justificatory knowledge for design science research, where the theory-driven derivation of the problem and solution space is fundamental (Gregor and Hevner 2013). Our validation steps also confirmed the potential value of our findings for future research, with one interviewee (S1, see Online Appendix 3) specifically highlighting that understanding the effects of DT is a prerequisite to study success factors of DE initiatives.

Second, considering that our findings are based on a high-level conceptualization of opportunity recognition, which integrates several theoretical perspectives, our study forms the basis from which it becomes possible to examine the *process* and *behavioral* perspectives in greater detail. More specifically, our findings draw from the four key constructs of opportunity recognition theory. We adopted the four constructs from earlier work on opportunity recognition from a process perspective in terms of *activities*, *input*, and *outcome*, e.g., Ardichvili et al. (2003). We consider the behavioral perspective to be implicitly represented in the construct *actor* along with its relationships. Starting from here, we can hypothesize which effects best support future research from a more detailed process and behavioral perspective of opportunity recognition: Effect #1 finds that and explains why there is a growing number and variety of *actors* engaging in the activities of the opportunity recognition process. Effects #2 and #3, by contrast, address how and why DT influences the input of opportunity recognition, i.e., *resource* and *market*, and thereby demonstrate why it is relevant to consider both the RBV and MBV when conceptualizing opportunity recognition in digital contexts. Finally, effects #4, #5, and #6 show how the DT concept expands the actor’s room for solutions which increases the scope and complexity of (traditional) activities for generating opportunity-ideas as the outcome of opportunity recognition. Thus, we argue that all presented effects relate to the process perspective in terms of *activities*, *input*, and *outcome*. Further, our results show that DT specifically influences the cognitive ability (i.e., effect #1) and behavior of an actor (i.e., effect #4) engaging in opportunity recognition, which is why effects #1 and #4 specifically relate to the behavioral perspective. This finding is consistent with both effects focusing on the *actor* who is at the core of the behavioral perspective.

Third, referring to Berger et al. (2019) who ask how DT creates “opportunity spaces for entrepreneurial action” (p. 7), our findings provide relevant insights into the creation of digital opportunity spaces and the role of *market* and *resource* constructs, which relate to the MBV and RBV as two established theories (Barney 1991; Porter and Stern 1999). The effects of DT demonstrate why organizations need to consider their internal resource base as well as the external market base to recognize digital opportunities. For instance, digital platforms provide access to shared external digital resources from professional partners, competitors, or even customers (Ciriello et al. 2018; Selander et al. 2013). Further, DT requires actors to increasingly collaborate with other market participants, e.g., driven by continuous stakeholder integration through connected products. Accelerated by increasing digital invasiveness, DT entails changes in the role of the customer, e.g., by evolving into prosumers. In this regard, we build on and extend existing work by Davidsson (2015) who – to the best of our knowledge – was the first aiming to unfold the vague opportunity concept. In particular, he identified the *actor* together with three other central constructs of opportunity, i.e., *opportunity confidence*, *new venture ideas* (i.e., *opportunity-idea*), and *external enablers*. *Opportunity confidence* relates to opportunity evaluation and is therefore outside the scope of this study. In turn, we draw from his thoughts regarding the *opportunity-idea* but consider *resource* and *market* as separate constructs due to their central role in literature on opportunity recognition (Ardichvili et al. 2003). Finally, we understand and examine DT as an (external) enabler of opportunity recognition (von Briel et al. 2021) that influences all other constructs. In doing so, we specifically address Davidsson’s (2015) call for conceptual development of the effects of external enablers across the venture creation process.

### Practical Implications

We validated and further developed the effects of DT by applying them to 34 real-world cases of DE initiatives and by conducting seven expert interviews with practitioners working in digital contexts. Both steps of our validation confirmed the effects’ real-world fidelity and offered ideas concerning the value of these effects for practitioners in the future. From a practical perspective, our work supports managers in understanding the relevant effects of DT on opportunity recognition. Along these lines, our findings provide two kinds of practical value (Moeini et al. 2019).

First, as recognizing opportunities is one of the most important activities of entrepreneurs (Ardichvili et al. 2003), doing so is even more decisive for success in the digital world, where DT blurs boundaries between customers, companies, products, and industries (Oberländer et al. 2021; Yoo et al. 2010). In this regard, practitioners can use our work to discover but also create opportunities more effectively as the effects of DT define the digital opportunity space available to organizations. For instance, practitioners can continuously monitor their internal and external environment regarding the effects of DT, e.g., by keeping track of new DTs or analyzing their resource base in order to discover so far unrecognized opportunities. Practitioners could also actively leverage individual effects by drawing from insights of the provided rationales as well as of the identified real-world cases demonstrating the effects. In doing so, they can understand why and how the effects occur and hence try to influence underlying dynamics, e.g., by intensifying collaboration with other market participants on digital platforms. Thus one interviewee, a head of IoT and asset management in the health care industry, stated: “I think the model is great because I think it's structured nicely and shows interdependencies, and I think that alone helps. I mean for research but also for practitioners who are somewhere in the digital entrepreneurship field, I think it always helps to be aware of these effects, simply to become creative and to think about what my next step is, what can I do to become more innovative in general.”.

Second, practitioners can use our study to improve knowledge creation regarding opportunity recognition and apply corresponding knowledge to their business processes and organizational structures. At the same time, the importance of knowledge for successful opportunity recognition in DE contexts has been confirmed in the literature, e.g., by Sahut et al. (2021) and Sussan and Acs (2017). Broadly speaking, the application of existing knowledge or the creation of knowledge drives opportunity recognition by fostering experimentation and subsequent innovation. In this regard, our validation demonstrated that the effects added insightful explanations to the real-world cases and can support knowledge creation, in particular regarding ongoing dynamics in the understudied initiation of DE initiatives. Thus, the effects might be a valuable analytical perspective to consider for evaluation processes of DE initiatives, which potentially enhances the success of current or upcoming initiatives. For instance, it might be interesting for project prioritization processes to consider which effects relate to which project and which of these effects have led to successful results in the past. Further, to facilitate practical usage, practitioners could develop (IT) artifacts based on our findings that support their decision processes regarding DE initiatives.

## Conclusion

In this study, we identified and explained the effects of DT on opportunity recognition. Like any research, our work has limitations, which, in turn, provide stimuli for future research. First, for our systematic assessment of publications, we focused on knowledge from the digital innovation domain which is already comparably mature with regards to DT. Although our work in this regard follows Recker and von Briel’s (2019) “opportunities for interdisciplinary conversations” (p. 4) in DE, we might have missed further relevant publications. In particular, future research could conduct a second structured literature review in the DE domain or other DT-related domains to generate a broader sample of studies relevant to opportunity recognition in a digital world, and to validate and enhance our findings. Second, we deliberately built on a high-level conceptualization of opportunity recognition and came to a broad understanding of DT as an enabler of entrepreneurial endeavors. Thus, our effects do not provide specific insights regarding all the different theoretical perspectives we drew from, i.e., process and behavioral perspectives, MBV and RBV, and regarding all the much more detailed constructs that have been studied in the literature concerning opportunity recognition, e.g., experience, learning, or creativity. However, research can use our results as a basis from where existing knowledge on these constructs with regard to opportunity recognition can be studied in digital contexts, e.g., which effects relate to the creativity of the actor when recognizing opportunities and how they influence the actors behavior. Third, although we validated the effects by applying them to real-world cases, our study was conducted primarily from a theoretical perspective. Further research can use our findings to generate predictive or even prescriptive knowledge, and to develop artifacts that explicitly guide practitioners engaging in opportunity recognition. Fourth, the validation of our study entails limitations as we relied on secondary data and also on a limited number and duration of expert interviews. Thus, further research might want to engage in validation with primary quantitative or qualitative data, e.g., following a case study approach, potentially targeting various industries and company types. In this context, we wish to highlight that while we believe our findings to be a theory for explaining, we did not conduct comprehensive empirical testing, which leaves this open to future research. Finally, future research can use our results from the coding process, e.g., the selective codes, to identify and develop interrelations for future sensemaking, e.g., effects mediating each other.

To conclude, recognizing opportunities in a digital world will gain importance, accelerated by network effects and current socio-economic developments. We believe that this study is theoretically and practically relevant and hope it provides fellow DE scholars with a foundation to advance research on opportunity recognition in digital contexts.

## Supplementary Information

Below is the link to the electronic supplementary material.

Supplementary file1 (PDF 351 kb)
