# Supplementary material for: The Effects of Digital Technology on Opportunity Recognition
Source: Bus Inf Syst Eng. 2022 Feb 3;64(1):47–67. doi: 10.1007/s12599-021-00733-9 (PMC8811742; doi:10.1007/s12599-021-00733-9)
Supplement: Supplementary file 1 — Supplementary file1 (PDF 351 kb) [file 12599_2021_733_MOESM1_ESM.pdf]

The Effects of Digital Technology on Opportunity Recognition

Thomas Kreuzer, Anna Lindenthal, Anna Maria Oberländer, Maximilian Röglinger

Business & Information Systems Engineering (2021)

Appendix (available online via http://link.springer.com)

Appendix (Online)

Appendix 1: Details on the Literature Review in Terms of Scope of our Study, and Data Selection and Coding

During the step *search and select*, we initially found 300 publications from Web of Science and 429 publications from the AIS eLibrary. After applying the described quality filters (i.e., focus on high impact journals and conferences) and checking for duplets, we derived a data set of 154 publications. We used a five-point Likert scale to give each of the 154 publications a relevance score regarding our study's scope.

Consistent with our research question, our scope includes the effects of DT on opportunity recognition, whereby we understand creating new economic activities as an entrepreneurial process, during which actors engage in opportunity recognition (Gustavsson and Ljungberg 2018). Consequently, we required relevant publications to provide insights regarding the effects of DT, opportunity recognition and their interplay. We aimed to exclude publications from our study that focus on a general technology perspective or on detailed cognitive processes of actors recognizing opportunities. Table A4 provides details on what is in scope and out of scope of our study (Gustavsson and Ljungberg 2018).

Table A4: Review Scope of our Study

In Scope	Out of Scope
Effects of DT based on a broad understanding of DT as an enabler of entrepreneurial activities (von Briel et al. 2021)	Effects of specific technologies, e.g., artificial intelligence, and of digital systems, e.g., ERP systems;<br>Understanding of DT in domains other than DE and digital innovation
Effects of DT as an external enabler, e.g., as infrastructure, that influences opportunity recognition (Davidsson, 2015; von Briel et al. 2021)	Effects of contextual and environmental factors (e.g., geographical, cultural, low interest rate environment), success factors (e.g., favorability), and hindering effects with a negative influence on opportunity recognition
Effects of DT on opportunity recognition in terms of the identified four key constructs of opportunity recognition, i.e., the *actor*, *resource*, *market*, and *opportunity-idea*,	Effects of DT on opportunity recognition in terms of much more detailed constructs that have been studied in the literature on opportunity recognition, e.g., experience, learning, or creativity.<br>Effects of DT on the other phases of the entrepreneurial process, e.g., opportunity evaluation and exploitation

By screening each publication's title, abstract, and keyword regarding the terms DT and opportunity recognition, we selected publications meeting our review scope and boundaries following Cram et al. (2016). Each publication was assigned a relevance score for meeting the review scope. 59 irrelevant publications (score = 1) were excluded. For the remaining publications, we rated their relevance from low (score = 2) to focus (score = 5) based on the full text. We rated a sample of 15 publications in the full author-team to align our understanding. Once aligned, one co-author then rated the remaining publications and discussed ambiguous ratings with the other co-authors. We included all (53) publications with a relevance score above two, added six more publications (6) with a relevance score of five from a forward and backward search, and ended with a final set of 59 publications to analyze.

Table A5 provides details on the Likert Scale we used to rate the relevance of the publications and the number of publications we rated for each score.

Table A5: Likert Scale Determining the Data Set

Score	Relevance	Explanation	#	Incl. in Data Set
1	Irrelevant	Term DT and opportunity recognition do not appear	59	No
2	Low	Term DT appears, but no focus on opportunity recognition	42	No
3	Medium	Term DT appears and weak connection to opportunity recognition	30	Yes
4	High	Focus on DT and opportunity recognition	18	Yes
5	Focus	Strong focus on DT and opportunity recognition and connection to the research question, i.e., the effects of DT on opportunity recognition	5+6 (forw./ backw. search)	Yes

Table A6 provides a complete overview of our set of literature, the corresponding foundation of DT, the corresponding coding of the key constructs of opportunity recognition, the corresponding coding of the direct and transitive effects of digital technology on opportunity recognition (in line with Figure 3) and the final selective codes (in line with A7).

To extend and substantiate explanatory insight on the six effects of DT on opportunity recognition, we conducted a second iteration of axial and selective coding. First, we attempted to structure and analyse the results of the first iteration, i.e., the effects and selective codes, by using prominent characterizations of DT, e.g., Yoo et al. (2010) and Benbya et al. (2020). Table A6 illustrates which publication in our data set draws from which study's characteristics of DT, i.e., Yoo et al. (2010), Yoo et al. (2012) or other publications. However, we realized that the characteristics of DT were inconsistently used in literature, could hardly be disentangled making it impossible to relate individual characteristics to effects, and studies mostly understood the DT concept as a general umbrella term (Baskerville et al. 2020; Denner et al. 2018). We found instead that DT influences opportunity recognition as a holistic enabler driven by three digital phenomena that build on DT outcomes through which the digital phenomena (mainly) emerged (see theoretical background section). To make our coding more transparent, we have included Table A7, which contains our final list of selective codes derived from both iterations of axial and selective coding. Each code is associated with at least one of the six effects in A7. In addition, Table A7 depicts each code's assignment to one of the key constructs of opportunity recognition (i.e., theory) and to the digital phenomena and DT outcomes (i.e., DT conceptualization) to show how the final list of selective codes represents the theory of opportunity recognition and DT conceptualization. The final list of selective codes is also depicted in Table A6 to inform which code can be found in which paper, to enhance transparency of our coding.

Table A6: Overview of Data Set of Papers and Codes of Direct and Transitive Effects of Digital Technology on Opportunity Recognition

Legend:<br>x = Directly Addressed<br>(x) = Indirectly Addressed			DT Conceptualization (Sources)				Key Constructs of Opportunity Recognition				Direct Effects of Digital Technology									Transitive Effects of Digital Technology						Selective Codes
	**Title**	**Source**	**Yoo et al. (2010)**	**Yoo et al. (2012)**	**Benbya et al. (2020)**	**Other Publication(s)**	**Actor**	**Market**	**Resource**	**Opportunity-Idea**	**1.1**	**1.2**	**1.3**	**2.1**	**2.2**	**2.3**	**3.1**	**3.2**	**3.3**	**4.1**	**4.2**	**5.1**	**5.2**	**6.1**	**6.2**	Reference to Selective Codes in Tabel A7 for the Direct and Transitive Effects of DT on Opportunity Recognition (After Second Iteration of Axial and Selective Coding)
1	The role of users and customers in digital innovation: Insights from B2B manufacturing firms	Abrell et al. (2016)	x	x			x	x	x	x	x	x			x	(x)	(x)	x	x	x	x	x	x	x	(x)	**1b, 1c, 1f, 1g, 1h, 1j, 2a, 2b, 2h, 3c, 3d, 4b, 4f, 4g, 4i, 4k, 5a, 5b, 5g, 6a, 6b, 6c, 6f, 6g, 6h, 6i**
2	Generating innovation potential: How digital entrepreneurs conceal, sequence, anchor, and propagate new technology	Arvidsson et al. (2018)	x				x		(x)	x	x	x		(x)	(x)	(x)				x		(x)				**1b, 1f, 1g, 1j, 2b, 2j, 4b, 4f, 4g, 4h, 4k, 5i**
3	Service innovation in the digital age: Key contributions and future directions	Barrett et al. (2015)	x	x			x	(x)	x	x	x	x	(x)	x	x	x			(x)	x	x			x	x	**1b, 1c, 1d, 1f, 1g, 1h, 2h, 2a, 2b, 2c, 2d, 3d, 5a, 5c,**
4	Social, commercial, or both? An exploratory study of the identity orientation of digital social innovations	Bonina et al. (2020)	x	x			x	x	x	x	x	x			x	(x)	(x)		x	x	x					**1b, 1c, 1f, 1g, 2i, 3e, 4b, 4f, 4g**
5	A Knowledge-Based Model of Radical Innovation in Small Software Firms	Carlo et al. (2012)				Lyytinen and Rose (2003)	x	(x)	x	x	x	x		x	x	x			(x)	x	x					**1b, 1c, 2b, 2c, 2d, 1j, 2f, 2g, 2j, 3d, 4b, 4d, 4k**
6	Idea Hubs as Nexus of Collective Creativity in Digital Innovation	Ciriello and Richter (2015)	x	x			x		(x)	x	x	x			(x)	x				x		(x)		(x)	x	**1a, 1b, 1c, 1f, 2d, 2j, 3f, 2h, 4b, 4f, 5h, 5i, 6c, 6d, 6e**
7	Scenario-Based Design Theorizing: The Case of a Digital Idea Screening Cockpit	Ciriello and Richter (2019)	x	x			x		x	x	x	x			x					(x)					x	**1a, 1b, 1c, 2g, 2j, 4b, 6b, 6d**
8	From Process to Practice: Towards a Practice-based Model of Digital Innovation	Ciriello et al. (2017)	x	x			x			(x)	x	x								(x)				(x)	(x)	**1b, 1c, 2a, 2f, 2k, 4f, 4g, 6e, 6f, 6g**
9	Digital Innovation	Ciriello et al. (2018)	x	x			x	x	x	x	x	x		x	x	x	x			x	x	x	x	(x)		**1b, 1c, 1c, 1e, 1f, 1g, 1g, 1h, 1i, 1j, 2a, 2b, 2d, 2e, 2f, 2g, 2h, 2j, 2k, 3a, 3b, 3f, 3g, 3h, 3i, 4a, 4b, 4c, 4d, 4e, 4f, 4g, 4h, 4i, 4j , 4k, 5a, 5b, 5d, 5f, 5g, 5i, 5j, 6b, 6e, 6f, 6g, 6i, 6j**

10	The paradoxical effects of digital artifacts on innovation practices	Ciriello et al. (2019)	**x**	**x**			**x**		**x**	**x**	**x**	**x**			**x**					**(x)**		**(x)**	**(x)**	**x**		**1b, 1c, 1e, 1j, 2d, 2e, 2h, 3a, 3e, 3h, 3i, 4a, 4b, 4e, 4k, 5b, 5d, 5g, 5j, 6d, 6j**
11	The Digital Workplace is Key to Digital Innovation	Dery et al. (2017)				-	**x**	**x**	**x**	**x**	**x**	**x**			**x**		**x**			**(x)**	**(x)**	**(x)**	**(x)**		**x**	**1a, 1b, 1c, 1g, 1f, 2a, 2j, 3b, 3e, 4h, 4f, 4h, 5e, 5f, 5i, 6c, 6d**
12	Information technology and innovation outcomes: is knowledge recombination the missing link?	Dong and Yang (2019)	**x**				**x**		**(x)**	**(x)**	**x**	**x**		**(x)**						**(x)**					**(x)**	**1b, 1c, 1f, 2b, 4b, 4c, 4d, 6f**
13	What Determines the Adoption of Digital Innovations by Digital Natives? – The Role of Motivational Affordances	Ebermann et al. (2016)	**x**	**x**			**x**		**x**	**(x)**	**x**	**x**		**x**	**(x)**	**x**				**(x)**					**(x)**	**1b, 1c, 1f, 2a, 2d, 2g, 6c**
14	Untangling Generativity: Two Perspectives on unanticipated change produced by diverse actors	Eck and Uebernickel (2016)	**x**	**x**			**x**		**x**	**x**	**x**	**x**		**x**		**(x)**				**(x)**		**x**		**x**		**1b, 1c, 4f, 4g, 2f, 2h, 5e, 5j, 6g**
15	Digital innovation as a fundamental and powerful concept in the information systems curriculum	Fichman et al. (2014)	**x**				**x**	**(x)**	**x**	**(x)**	**x**	**x**			**x**	**x**		**(x)**	**(x)**	**(x)**	**(x)**	**(x)**	**(x)**		**(x)**	**1b, 1c, 2a, 2b, 2c, 2j, 3b, 3h, 4b, 4h, 5a, 5c, 5i, 6c, 6g,**
16	Initiating Ambidexterity through Digital Innovation Labs	Göbler et al. (2020)	**x**				**x**		**(x)**	**(x)**	**x**	**x**			**(x)**	**(x)**				**(x)**						**1b, 1c, 1f, 2d, 4a**
17	Entrepreneurship in the Digital Society	Gustavsson and Ljungberg (2018)	**x**	**x**			**x**	**(x)**	**x**	**x**	**x**	**x**			**x**				**(x)**	**x**	**x**	**x**	**x**		**(x)**	**1b, 1c, 1j, 2d, 2h, 2j, 3h, 4b, 4c, 4d, 4k, 5a, 5b, 5d, 5g, 5i, 6g,**
18	Architecting Structural Flexibility in Design Processes – a Case Study of Public Sector Digital Innovation	Hedlund (2019)	**x**	**x**			**x**		**x**		**x**			**x**		**(x)**										**1b, 2b, 2d, 2j**
19	Recombination in the open-ended value landscape of digital innovation	Henfridsson et al.(2018)	**x**	**x**			**x**	**x**	**x**	**x**	**x**	**x**	**x**	**x**		**x**	**x**			**x**	**x**	**x**	**x**		**(x)**	**1a, 1b, 1c, 1d, 1e, 1i, 1j, 2j, 3f, 3h, 4a, 4b, 4c, 4d, 4e, 4j, 4k, 5a, 5d, 5f, 5g, 5i, 5j, 6e, 6g**
20	Looking for a Few Good Concepts and Theories for Digitized Artifacts and Digital Innovation in a Material World	Herterich et al. (2016)	**x**	**x**			**x**		**x**	**(x)**	**x**			**x**	**x**	**x**				**(x)**	**(x)**	**(x)**		**(x)**		**1b, 1f, 1h, 1i, 1j, 2b,2d, 2g, 4b, 4f, 4g, 4i, 4j, 4k, 6j**
21	Digital innovation and transformation: An institutional perspective	Hinings et al. (2018)	**x**	**x**			**x**	**(x)**	**(x)**	**(x)**	**x**						**(x)**	**(x)**	**x**						**x**	**1b, 1f, 3g, 3i, 6c, 6j**
22	Temporal Ambidexterity: How Digital Innovation Labs Connect Exploration and Exploitation for Digital Innovation	Holotiuk and Beimborn (2019)	**x**	**x**			**x**	**(x)**	**(x)**	**x**	**x**	**x**			**(x)**	**(x)**	**(x)**			**(x)**				**(x)**	**(x)**	**1b, 1f, 2d, 2g, 3i, 4g, 6f, 6j**
23	The current state and future opportunities of digital innovation: A literature review	Hund et al. (2019)	**x**	**x**			**x**		**x**	**x**	**x**	**x**			**(x)**			**(x)**	**(x)**	**x**	**x**				**(x)**	**1b, 1e, 1h, 1i, 1j, 2a, 2h, 3c, 3d, 3f, 3h, 3i, 4b, 4c, 4d, 4e, 4i, 4j, 4k, 6j**

24	Knowledge Management in the Digital Era: How Digital Innovation Labs Facilitate Knowledge Recombination	Hund et al. (2019b)	x	x			x		(x)	(x)	x			x		(x)				x	x	(x)	(x)	(x)	x	1a, 1b, 1f, 1h, 1i, 2a, 2b, 2j 4b, 4h, 4i, 5b, 5c, 5i, 6f, 6h, 6i, 6j
25	EXPLORING PHRONESIS IN DIGITAL INNOVATION	Hylving and Koutsikouri (2020)	x	x			x			(x)	x				(x)					(x)		(x)	(x)	(x)		1b, 1f, 2j, 2h, 2k, 5g, 5i, 5j, 6h, 6j
26	The Future Digital Innovators: Empowering the Young Generation with Digital Fabrication and Making	Iivari et al. (2016)				Davies 2011; Eshet-Alkalai 2004; Ezziane 2007	x	x	x	(x)	x	x							(x)	(x)				(x)		1b, 1f, 3e, 3i, 4f, 6c, 6j
27	Fostering digital innovation through inter-organizational collaboration between incumbent firms and start-ups	Islam et al. (2017)	x				x	x		(x)	x	x		x				x						(x)		1b, 1f, 2f, 3e, 6c, 6d
28	Digital innovation: A review and synthesis	Kohli and Melville (2019)	x				x	x		(x)	x	x		x					(x)	(x)					(x)	1b, 1f, 2a, 2b, 2j, 3f, 3g, 6f
29	Digital Innovation and the Division of Innovative Labor: Digital Controls in the Automotive Industry	Lee and Berente (2012)	x				x	x	x	(x)	x			x				x		(x)	(x)	x	x			1b, 1f, 2a, 3b, 4b, 4h, 5a, 5b, 5j
30	Offerings that are “Ever-in-the-Making”: Post-Launch Continuous Digital Innovation in Late-Stage Entrepreneurial Ventures	Lehmann and Recker (2019)	x	x			x		x	x	x				x	x		x	(x)	(x)	(x)	(x)			(x)	1b, 1h, 1i, 1j, 2e, 2j, 2k, 3b, 3f, 4b, 4c, 4i, 4j, 4k, 5a, 5b, 5c, 5g, 5i, 5j, 6c, 6e, 6i
31	Does One Size Fit All? Theorizing Governance Configurations for Digital Innovation	Leonhardt et al. (2018)	x	x			x	x	x	(x)	x				x	(x)				x	x					1b, 1h, 1j, 2b, 2g, 4i, 4k
32	Organizational readiness for digital innovation: Development and empirical calibration of a construct	Lokuge et al. (2019)	x	x			x	x	x	x	x	x		x	x	x	x	x		(x)						1b, 1c, 1f, 2b, 2d, 2f, 3a, 3b, 3c, 3f, 3g, 3h, 4b, 4g
33	Service innovation: A service-dominant logic perspective	Lusch and Nambisan (2015)	x				x		x	(x)	x	x		x	x	x		x		x	x	(x)	(x)	x	(x)	1b, 1f, 1h, 1i, 2b, 2d, 2j, 3b, 3c, 3e, 3i, 4b, 4i, 4j, 5e, 5f, 6c, 6d, 6i, 6j
34	A Framework for Recognizing Digital Transformation Opportunities	Muehlburger et al. (2020)		x			x		x	(x)	x				(x)	x				(x)		(x)	(x)			1b, 1f, 1g, 2a, 2g, 4h, 5a, 5b, 5f
35	Managing Ambidexterity in Startups Pursuing Digital Innovation	Mueller et al. (2019)	x	x			x		x	x	x	x		(x)		x	(x)							(x)		1b, 1f, 1g, 2a, 2b, 3i, 5a
36	Architecture vs. ecosystem perspectives: Reflections on digital innovation	Nambisan (2018)	x				x		x	x	x				x		(x)			x	x					1b, 1c, 2d, 3i, 4b, 4c, 4d

37	Digital Innovation Management: Reinventing Innovation Management Research in a Digital World	Nambisan et al. (2017)	**x**				**x**	**(x)**	**x**	**(x)**	**x**	**x**		**(x)**	**x**	**x**						**x**	**x**		**(x)**	**1b, 1c, 1f, 1h,2b, 2h, 2j, 5a, 5g, 5i 6h, 6j**
38	Trajectory Dynamics in Innovation: Developing and Transforming a Mobile Money Service Across Time and Place	Oborn et al. (2019)	**x**	**x**			**x**		**(x)**	**(x)**	**x**			**(x)**	**x**			**(x)**	**(x)**			**(x)**				**1b, 1c, 2a, 2b, 2d,2h, 2j, 3a, 3b, 3e, 3f, 3g, 3h, 3i. 5g, 5i**
39	Utilising the innovation potential - A systematic literature review on employee-driven digital innovation	Opland et al. (2020)	**x**	**x**			**x**	**(x)**	**x**	**x**	**x**	**x**			**(x)**					**(x)**				**(x)**		**1a, 1b, 2a, 2j, 4b, 4h, 6f, 6h, 6j**
40	Digital Innovation, Platform Orientation and the Performance of IT Startups	Oppong-Tawiah and Bassellier (2017)	**x**	**x**			**x**	**(x)**	**(x)**		**x**	**x**		**x**	**x**	**(x)**	**(x)**			**(x)**				**x**	**(x)**	**1b, 1g, 1i, 1j, 2a, 2b, 2j, 2k, 3b, 3h, 3i, 4j, 4k, 6a, 6c, 6i, 6j**
41	The Future of Digital Entrepreneurship Research: Existing and Emerging Opportunities	Recker and von Briel (2019)	**x**				**x**	**(x)**	**(x)**		**x**		**(x)**	**(x)**	**(x)**	**(x)**	**(x)**		**(x)**							**1b, 1c, 1h, 1i, 2c, 2d, 2e, 2f, 2j, 2k, 3b, 3h, 3i**
42	A Work Model for Employee-Driven Innovation in Public Organizations	Reibenspiess et al. (2019)	**x**				**x**	**x**	**x**	**x**	**x**	**x**		**(x)**	**(x)**				**(x)**	**(x)**		**(x)**	**(x)**			**1a, 1b, 1j, 2b, 2j, 3e, 3d, 3g, 4i, 4k, 5a, 5i**
43	An integrated model of innovation drivers for smaller software firms	Rose et al. (2016)				Rose (2010); Carlo et al. (2011)	**x**	**x**		**(x)**	**x**	**x**			**x**		**x**	**(x)**		**x**		**(x)**	**x**	**(x)**		**1b, 1f, 1j, 2j, 2k, 3c, 3g, 3i, 4k, 5i, 5j, 6c, 6f**
44	The age of digital entrepreneurship	Sahut et al. (2021)	**x**				**x**	**x**	**x**	**x**	**x**	**(x)**		**(x)**	**(x)**	**(x)**			**(x)**					**(x)**	**(x)**	**1b, 1h, 2b, 2d, 2g, 2j, 3d, 3h, 3i, 6g, 6j**
45	Leveraging Customer Involvement for Fueling Innovation: The Role of Relational and Analytical Information Processing Capabilities	Saldanha et al. (2017)	**x**	**x**			**x**	**x**	**x**		**x**	**x**		**x**	**x**	**(x)**	**(x)**	**x**						**x**		**1b, 1c, 1g, 2a, 2b, 3c, 3h, 3i, 6c, 6g**
46	Linking information systems and entrepreneurship: A review and agenda for IT-associated and digital entrepreneurship research	Steininger (2019)				Beck et al. (2017); Lyytinen and Rose (2003)	**x**	**x**	**x**	**(x)**	**x**			**(x)**				**x**					**(x)**			**1b,1f, 2d, 2g, 3c, 4b, 5d, 5f**
47	Assessing value creation in digital innovation ecosystems: A Social Media Analytics approach	Suseno et al. (2018)	**x**				**x**	**x**	**x**	**(x)**	**x**			**x**		**(x)**			**x**	**x**				**x**		**1b, 1e, 1h, 2b, 2h, 2j, 3b, 3e, 3f, 3h, 3i, 4b, 4e, 4f, 4g, 6c, 6h, 6i**
48	A Threesome Dance of Agency: Mangling the Sociomateriality of Technological Regimes in Digital Innovation	Svahn et al. (2009)				Boland et al. (2007); Yoo et al. (2008)	**x**	**x**	**x**		**x**	**x**		**x**	**x**	**(x)**		**x**								**1b, 1f, 1g, 1h, 1i, 2a, 2b, 2f, 2g, 2h, 2j, 2k, 3b, 3c, 3f, 3h, 3i**

49	Embracing Digital Innovation in Incumbent Firms: How Volvo Cars Managed Competing Concerns	Svahn et al. (2017)	**x**	**x**			**x**	**(x)**	**(x)**	**(x)**	**x**	**x**		**x**	**x**	**(x)**		**x**		**x**	**x**	**x**	**(x)**	**(x)**		**1b, 1f, 1h, 1j, 2b, 2e, 2j, 2k, 3b, 3e, 3i, 4b, 4c, 4d, 4i, 4k, 5a, 5c, 5f, 5i, 5j, 6d, 6i, 6j**
50	Service-Dominant Business Model Design for Digital Innovation in Smart Mobility	Turetken et al. (2019)				Alam et al. (2016)	**x**	**x**	**(x)**	**(x)**	**x**				**x**			**x**		**(x)**				**(x)**		**1b, 1f, 1j, 2c, 2f, 2i, 2j, 3b, 3f, 4g, 4h, 4k, 6c, 6e**
51	A comprehensive framework to research digital innovation: The joint use of the systems of innovation and critical realism	Vega et al. (2019)	**x**	**x**			**x**	**x**	**x**	**x**	**x**	**x**		**x**	**x**			**x**	**x**							**1b, 1f, 1h,1j, 2b, 2j, 2k, 3f, 3h, 3i**
52	Researching Digital Entrepreneurship: Current Issues and Suggestions for Future Directions	von Briel et al. (2021)	**x**				**x**		**x**	**(x)**	**x**	**(x)**		**(x)**	**(x)**	**(x)**			**(x)**	**(x)**	**(x)**	**(x)**	**(x)**			**1b, 1f, 1h, 2b, 2d, 2h, 2j, 2k, 3b, 3f, 3h, 3i, 4b, 4e, 5a, 5d, 5i, 5f**
53	How Do Community Ecology and Structure Shape Digital Innovation Strategy?	Wang et al. (2016)	**x**	**x**			**x**	**(x)**	**x**	**x**	**x**	**x**				**(x)**		**(x)**	**x**	**(x)**		**(x)**	**(x)**	**(x)**		**1c, 2g, 3b, 3d, 3f, 3h, 3i, 4b, 5a, 5d, 5j, 6g, 6j**
54	The Impact of IOS Use and Interpersonal Ties on Digital Innovation: Insights from Boundary Spanning and Institutional Theories	Wei et al. (2019)	**x**				**x**	**(x)**		**x**	**x**	**x**		**x**				**x**	**(x)**	**x**						**1b, 1f, 2b, 2j, 3b, 3c, 3g, 4d**
55	Innovating in a digital world-the role of digital product innovation capabilities.	Wiesböck (2019)	**x**	**x**			**x**	**x**		**(x)**	**x**				**x**	**x**				**(x)**	**(x)**					**1b, 1f, 2b, 2d, 2j, 4b, 4g**
56	Digital innovations Embedding in organizations	Wiesböck et al. (2019)	**x**	**x**			**x**	**x**	**x**	**(x)**	**x**	**x**		**x**	**x**	**(x)**		**x**		**(x)**	**(x)**					**1b, 1f, 2b, 2d, 2h, 2j, 3b, 3c, 4b, 4g**
57	The New Organizing Logic of Digital Innovation: An Agenda for Information Systems Research	Yoo et al. (2010)	**x**				**x**	**x**	**x**	**x**	**x**	**x**		**x**	**x**	**x**		**x**		**x**	**x**	**x**	**x**	**(x)**	**(x)**	**1b, 1e, 1f, 1h, 1i, 1j, 2a, 2b, 2c, 2d, 2e, 2f, 2h, 2j, 2k, 3b, 3f, 3h, 3i, 4a, 4b, 4e, 4i, 4j, 4k, 5a, 5b, 5c, 5d, 5g, 5i, 5j, 6b, 6c, 6e, 6g**
58	Organizing for Innovation in the Digitized World	Yoo et al. (2012)	**x**	**x**			**x**	**x**	**(x)**	**x**	**x**	**x**	**(x)**	**x**	**x**	**(x)**		**(x)**	**(x)**	**x**	**x**	**x**	**x**	**(x)**		**1b, 1c, 1e, 1f, 1h, 1i, 1j, 2a, 2b, 2c, 2d, 2e, 2f, 2h, 2j, 2k, 3b, 3f, 3h, 3i, 4a, 4b, 4e, 4i, 4j, 4k, 5a, 5b, 5c, 5d, 5g, 5i, 5j, 6b, 6e, 6g, 6j**
59	Digital Innovation in Industrial-Age Firms: Managing the Balancing Act of Knowledge Integration	Zapadka (2020)	**x**	**x**			**x**	**(x)**	**(x)**	**x**	**x**	**x**				**(x)**	**(x)**	**(x)**		**(x)**		**(x)**	**x**	**(x)**		**1b, 1f, 1e, 1h, 2h, 2j, 2k, 3b, 3c, 3i, 4b, 4i, 5g, 5i, 5j, 6f, 6i**

Table A7: Selective Codes for the Direct and Transitive Effects of DT on Opportunity Recognition (After Second Iteration of Axial and Selective Coding)

	#	From	To	Driven by	Through	Final List of Selective Codes (After Second Iteration) (ID)										
**Direct Effects**	**1**	Homogenous entrepreneurs	Growing number and variety of actors (everyone)	Increasing digital invasiveness	Layered architecture	Employees as new actors (1a)	Heterogenous actors engaging in opportunity recognition (1b)	Self-organizing new actors (1c)	Software agent as an actor (1d)	Convergence of DT (1e)	Digital invasiveness of private and professional life (1f)	Digital invasiveness enhancing digital capabilities of everyone (1g)	High diffusion of DT enabled by layered architecture (1h)	Layered modular architecture of loosely coupled components (1i)	Standardized protocols and interfaces (APIs) (1j)	
	**2**	Exclusively internal access only	Externally shared access	Dissolving company and customer boundaries	Digital platforms	New digital capabilities (2a)	Shared access to external resources (2b)	Shared digital assets (2c)	Transformed resource bases of organizations (2d)	Changes after deployment across company and customer boundaries (2e)	Democratized access to DT (2f)	Digital value networks across company and customer boundaries (2g)	Generativity of DT (2h)	Open data (2i)	Digital Platforms (2j)	Modification of digital artifacts (2k)
	**3**	Hierarchical relationships	Multi-lateral value networks	Dissolving product and industry boundaries	Digital ecosystems	Competitors becoming partners (3a)	DT fostering collaboration with other market Participants (3b)	Integration of suppliers (3c)	Regulatory changes regarding DT (3d)	Co-creation strategies (3e)	Convergence of DT dissolving product and industry boundaries (3f)	DT fostering knowledge sharing (3g)	Digital infrastructure across product and industry boundaries (3h)	Digital ecosystems (3i)		
**Transitive Effects**	**4**	Context-dependent restrictions	Multitude of (re-) combination possibilities	Increasing digital invasiveness	Layered architecture	New possibilities to combine data (4a)	New possibilities to combine resources (4b)	Re-combination in design (4c)	Re-combination in use (4d)	Convergence of DT (4e)	Digital invasiveness enabling high affordability of DT (4f)	Digital invasiveness enabling high availability, of DT (4g)	Digital invasiveness enhanced digital capabilities of everyone (4h)	High diffusion of DT enabled by layered architecture (4i)	Layered modular architecture of loosely coupled components (4j)	Standardized protocols and interfaces (APIs) (4k)
	**5**	Deterministic and final deployment	Continuous iterative development	Dissolving company and customer boundaries	Digital platforms	Continuous improvement of digital Offerings (5a)	Ever-present possibility to change and improve software (5b)	Iterative development of digital innovation (5c)	Changes after deployment across company and customer boundaries (5d)	Democratized access to DT (5e)	Digital value networks across company and customer boundaries (5f)	Generativity of DT (5g)	Open data (5h)	Digital Platforms (5i)	Modification of digital artifacts (5j)	
	**6**	Few occasion-related interactions	Continuous integration	Dissolving product and industry boundaries	Digital ecosystems	DT fostering customer Integration (6a)	DT fostering the continuous exchange of information (6b)	Simplified involvement of users and customers (6c)	Co-creation strategies (6d)	Convergence of DT dissolving product and industry boundaries (6e)	DT fostering knowledge sharing (6f)	Digital Infrastructure across product and industry boundaries (6g)	Short product cycles for the evolvement of digital Offerings (6h)	Continuous evolvement of digital offerings (6i)	Digital Ecosystems (6j)	

Legend: [To] Selective Codes; [Driven by | Through] Selective Codes

Appendix 2: Real-World Cases of DE Initiatives

For data collection, we performed a structured case search focusing on Urbach and Röglinger's (2019) collection of digitalization cases (21 cases), one of the leading international practitioner-oriented journal MISQE (11 cases), and the handbook of digital innovation by Nambisan et al. (2020) (2 cases). We used the search string ("digital innovation" AND "case") and limited our search to the period till January 31, 2021. Initially, we found 59 cases by following the understanding of DE of Briel et al. (2021) and Yoo et al. (2010). After excluding 25 cases that did not provide enough information, our final data set comprised 34 cases.

Table A8 provides the complete compilation of the 34 real-world Cases of DE initiatives we used to gain further insights on and validate our effects. Following our coding process, each case is associated with at least one or more effects of DT on opportunity recognition.

Table A8: Cases of DE Initiatives and Associated Effects of Digital Technology

Legend:<br>x = Case Refers to Effect				**Effects of DT on Opportunity Recognition**					
**Real-World Case**	**Authors**	**Organization**	**DT/<br>Digital Artifact**	**1**	**2**	**3**	**4**	**5**	**6**
**Urbach, Röglinger 2019 – Digitalization Cases<br>Part I Digital Disruption**									
Case1:<br>Enabling Digital Transformation Through Robotic Process Automation at Deutsche Telekom	Manfred Schmitz, Christian Dietze, and Christian Czarnecki	**Deutsche Telekom**	**Robotic Process Automation**					**x**	
Case 2:<br>Airline Application Security in the Digital Economy: Tackling Security Challenges for Distributed Applications in Lufthansa Systems	Balázs Somoskői, Stefan Spahr, Erkuden Rios, Oscar Ripolles, Jacek Dominiak, Tamás Cserveny, Péter Bálint, Peter Matthews, Eider Iturbe, and Victor Muntés-Mulero	**Lufthansa**	**Cloud Computing**			**x**			**x**

Case 3:<br>Digital Technologies for Ordering and Delivering Fashion: How Baur Integrates the Customer's Point of View	Daniel Baier, Alexandra Rese, Nikita Nonenmacher, Steve Treybig, and Benjamin Bressem	**Baur**	**Website**						**x**
Case 4:<br>Applying Sound-Based Analysis at Porsche Production: Towards Predictive Maintenance of Production Machines Using Deep Learning and Internet-of-Things Technology	Matthias Auf der Mauer, Tristan Behrens, Mahdi Derakhshanmanesh, Christopher Hansen, and Stefan Muderack	**Porsche Production**	**Predictive Maintenance, Deep Learning, IoT**	**x**			**x**	**x**	
**Urbach, Röglinger 2019 – Digitalization Cases**<br>**Part II Digital Business**									
Case 5:<br>Industry 4.0 Enabling Smart Air: Digital Transformation at KAESER COMPRESSORS	Maximilian Bock, Martin Wiener, Ralf Gronau, and Andreas Martin	**Kaeser Compressor**	**Industry 4.0 Technologies**						**x**
Case 6:<br>Dual-Track's Strategy for Incumbent's Transformation: The Case of Danske Bank Adopting a Platform Business Model	Kalina S. Staykova and Jan Damsgaard	**Danske Bank**	**Mobile Pay as Digital Platform**	**x**	**x**	**x**		**x**	**x**
Case 7:<br>Digitalization Partnership: How GKN Established a Digital Platform with 3YD to Realize the Disruptive Potential of Metal Additive Manufacturing	Klaus Wildhirt, Claudius Seidel, Udo Bub, Markus Josten, and Stephan Kühr	**GKN**	**3D Printing, Digital Paltform**	**x**	**x**	**x**			**x**
Case 8:<br>Socio-technical Complexity in Digital Platforms: The Revelatory Case of Helix Nebula: The Science Cloud	Michael Blaschke	**Helix Nebula**	**Digital Platforms and Cloud Services**		**x**	**x**			
Case 9:<br>Sitecore: Retaining Technological Leadership Through Digital Tech Acquisitions	Stefan Henningsson and Nishu Nishu	**Sitecore**	**Digital Plattform**		**x**			**x**	
Case 10:<br>Development of Strategies and Transformation Paths for Structured and Targeted Digital Change: The Case of the Presbyterian Church of Ghana Trinity Congregation	Sylvester Tetey Asiedu and Richard Boateng	**Presbyterian Church of Ghana Trinity Congregation**	**Webtechnology**	**x**		**x**			**x**

Case 11:<br>Creating a Digital Consulting Solution for Project Management Assessments at Dr. Kuhl Unternehmensberatung: Development and Initial Demonstration of a Fully Automated Asset-Based Consulting Approach	Volker Nissen, Jochen Kuhl, Hendrik Kräft, Henry Seifert, Jakob Reiter, and Jim Eidmann	**Dr. Kuhl Unternehmensberatung**	**Webtechnology**	**x**		**x**			
Case 12:<br>Building a Digitally Enabled Future: An Insurance Industry Case Study on Digitalization	Janina Weingarth, Julian Hagenschulte, Nikolaus Schmidt, and Markus Balser	**INSUR**	**Digital Technology**	**x**		**x**			
**Urbach, Röglinger 2019 – Digitalization Cases**<br>**Part III Digital Transformation**									
Case 13:<br>Digital Transformation of ABB Through Platforms: The Emergence of Hybrid Architecture in Process Automation	Johan Sandberg, Jonny Holmström, and Kalle Lyytinen	**ABB**	**Internet of Things, Digital Platforms and Ecosystems**		**x**	**x**			**x**
Case 14:<br>Digitalization of Information-Intensive Logistics Processes to Reduce Production Lead Times at ENGEL Austria GmbH: Extending Value Stream Mapping with Subject-Oriented Business Process Management	Christoph Moser and Karel Říha	**ENGEL**	**ERP Systems**	**x**			**x**	**x**	**x**
Case 15:<br>Digitalization in Public Services: Process Automation and Workforce Management at BruderhausDiakonie, a Social Services Organization	Ulrich Müller and Thomas Deelmann	**Bruderhaus Diakonie**	**ServiceNow as IT automation platform**	**x**	**x**	**x**			
Case 16:<br>Digital Health Innovation Engineering: Enabling Digital Transformation in Healthcare: Introduction of an Overall Tracking and Tracing at the Super Hospital Aarhus Denmark	Sven Meister, Anja Burmann, and Wolfgang Deiters	**Super Hospital Aarhus Denmark**	**Digital Technology**	**x**			**x**	**x**	
Case 17:<br>Digital Transformation in Healthcare: How the Potential of Digital Health Is Tackled to Transform the Care Process of Intensive Care Patients Across All Healthcare Sectors	Charlotte Vogt, Martin Gersch, Claudia Spies, and Konrad Bengler	**German Federal Ministry of Education and Research**	**Digital Technology**	**x**			**x**		
Case 18:<br>Data Innovation @ AXA Germany: Journey Towards a Data-Driven Insurer	Alexa Scheffler and Christian Paul Wirths	**Axa**	**Data Analytics**	**x**	**x**	**x**			**x**

Case 19:<br>Volkswagen Education Lab: Accelerating the Digital Transformation of Corporate Learning	Mathias Wildgrube, Nils Schaupensteiner, and Jan Wehinger	**Volkswagen**	**Robotic, AI, Virtual Reality**	x		x			
Case 20:<br>Navigating Through Digital Transformation Using Bimodal IT: How Changing IT Organizations Facilitates the Digital Transformation Journey at Deutsche Bahn Vertrieb GmbH	Lea Fortmann, Ingmar Haffke, and Alexander Benlian	**Deutsche Bahn Vertrieb GmbH**	**Bimodal IT, IT Systems**	x					
Case 21:<br>How the US Federal Communications Commission Managed the Process of IT Modernization	Kevin C. Desouza, James S. Denford, and Rashmi Krishnamurthy	**US Federal Communications Commission**	**Cloud Computing**	x					x
**Nambisan, Lyytinen, and Yoo (2020) – Handbook of Digital Innovation**<br>**Part II: Foundations of Digital Innovation**									
Chapter 3: Digital Innovation and Entrepreneurship in and around Institutional Voids: The Case of Fundación Cardiovascular (FCV) in Colombia	Sirkka L. Jarvenpaa, Elsie L. Echeverri-Carroll and Aura Pedraza-Avella	**Fundación Cardiovascular (FCV)**	**Telemedicine Services**	x	x	x	x	x	x
Chapter 4: Theorizing the Connections between Digital Innovations and Societal Transformation: Learning from the Case of M-Pesa in Kenya	M. Lynne Markus and Wenxiu (Vince) Nan	**M-Pesa**	**Mobile Payment via App and Mobile Money**	x			x	x	x
**MIS Quarterly Executive**<br>**Search String: “digital innovation” AND “case”**									
How Companies Can Benefit from Interlinking External Crowds and Internal Employees	Volkmar Mrass, Christoph Peters, Jan Marco Leimeister	**Airbus/ Local Motors**	**Digital Technology**	x	x	x	x		x
How AUDI AG Established Big Data Analytics in Its Digital Transformation	Christian Dremel, Matthias Herterich, Jochen Wulf, Jean-Claude Waizmann, Walter Brenner	**Audi**	**Big Data**	x					x
How AUDI AG is Driving Toward the Sharing Economy	Martin Mocker, Nils O. Fonstad	**Audi**	**Sharing Economy (Car Sharing)**	x	x			x	x
APC Forum: Leveraging Emerging Digital Technology at BP	Madeline Weiss, Heather A. Smith	**BP**	**Digital Technology**			x	x		
How DBS Bank Pursued a Digital Business Strategy	Siew Kien Sia, Christina Soh, Peter Weill	**DBS**	**Digital Technology**	x	x	x	x	x	
Cognitive Automation as Part of Deakin University's Digital Strategy	Mary C. Lacity, Rens Scheepers, Leslie P. Willcocks	**Deakin University**	**Cognitive Automation**	x	x			x	x
How GoGet CarShare's Product-Service System is Facilitating Collaborative Consumption	Felix Ter Chian Tan, Michael Cahalane, Barney Tan, Jonathan Englert	**Go Get Car Share**	**Product-Service-Systems**	x	x	x		x	x

Data Analytics Contributes to Better Decision-Making Beyond Organizational Boundaries	Wenyu (Derek) Du, Shan L. Pan, Kang Xie, Jinghua Xiao	**GSE**	**Data Analytics**		**x**	**x**			
Hummel's Digital Transformation Toward Omnichannel Retailing: Key Lessons Learned	Rina Hansen, Sia Siew Kien	**Hummel**	**Omnichannel Retailing**	**x**	**x**	**x**	**x**	**x**	**x**
How LEGO Built the Foundations and Enterprise Capabilities for Digital Leadership	Omar El Sawy, Henrik Amsinck, Pernille Kraemmergaard, Anders Lerbech Vinther	**LEGO**	**Capabilities for Digital Leadership**	**x**	**x**	**x**	**x**	**x**	**x**
How USAA Architected its Business for Life Event Integration	Martin Mocker, Jeanne Ross, Craig Hopkins	**USAA**	**Digital Technology**	**x**	**x**				

Real-world Cases – References

Dremel, Christian; Herterich, Matthias; Wulf, Jochen; Waizmann, Jean-Claude; and Brenner, Walter (2017) "How AUDI AG Established Big Data Analytics in Its Digital Transformation," *MIS Quarterly Executive*: Vol. 16 : Iss. 2 , Article 3.

Du, Wenyu (Derek); Pan, Shan L.; Xie, Kang; and Xiao, Jinghua (2020) "Data Analytics Contributes to Better Decision-Making Beyond Organizational Boundaries," *MIS Quarterly Executive*: Vol. 19 : Iss. 2 , Article 5.

El Sawy, Omar; Amsinck, Henrik; Kraemmergaard, Pernille; and Lerbech Vinther, Anders (2016) "How LEGO Built the Foundations and Enterprise Capabilities for Digital Leadership," *MIS Quarterly Executive*: Vol. 15 : Iss. 2 , Article 5.

Hansen, Rina and Kien, Sia Siew (2015) "Hummel's Digital Transformation Toward Omnichannel Retailing: Key Lessons Learned," *MIS Quarterly Executive*: Vol. 14 : Iss. 2 , Article 3.

Lacity, Mary C.; Scheepers, Rens; and Willcocks, Leslie P. (2018) "Cognitive Automation as Part of Deakin University's Digital Strategy," *MIS Quarterly Executive*: Vol. 17 : Iss. 2 , Article 4.

Mocker, Martin and Fonstad, Nils O. (2017) "How AUDI AG is Driving Toward the Sharing Economy," *MIS Quarterly Executive*: Vol. 16 : Iss. 4 , Article 4.

Mocker, Martin; Ross, Jeanne; and Hopkins, Craig (2015) "How USAA Architected its Business for Life Event Integration," *MIS Quarterly Executive*: Vol. 14 : Iss. 4 , Article 6.

Nambisan, S., Lyytinen, K., & Yoo, Y. (Eds.) (2020). Research handbooks in business and management series. Handbook of digital innovation. Cheltenham, UK, Northampton, MA: Edward Elgar Publishing.

Sia, Siew Kien; Soh, Christina; and Weill, Peter (2016) "How DBS Bank Pursued a Digital Business Strategy," *MIS Quarterly Executive*: Vol. 15 : Iss. 2 , Article 4.

Tan, Felix Ter Chian; Cahalane, Michael; Tan, Barney; and Englert, Jonathan (2017) "How GoGet CarShare's Product-Service System is Facilitating Collaborative Consumption," *MIS Quarterly Executive*: Vol. 16 : Iss. 4 , Article 5.

Urbach, N., & Röglinger, M. (Eds.) (2019). Management for professionals. Digitalization cases: How organizations rethink their business for the digital age. Cham, Switzerland: Springer.

Weiss, Madeline and Smith, Heather A. (2008) "APC Forum: Leveraging Emerging Digital Technology at BP," *MIS Quarterly Executive*: Vol. 6 : Iss. 2 , Article 6.

Appendix 3: Expert Interviews

During the *VALIDATION* stage, we aimed at validating the *real-world fidelity, completeness, internal consistency,* and *level of detail* of our effects conceptualizing the effects of DT on opportunity recognition (Sonnenberg and vom Brocke 2012). To do so, we followed two steps: (1) Application of the effects to secondary data of real-world Cases of DE initiatives, and (2) semi-structured expert interviews with entrepreneurship and innovation scholars in the IS domain and practitioners working in digital context.

First, we applied our effects to secondary data of 34 real-world Cases of DE initiatives and thereby gained initial insights regarding its *real-world fidelity* and *completeness*. For data collection, we performed a structured case search focusing on Urbach and Röglinger's (2019) collection of digitalization cases (21 cases), one of the leading international practitioner-oriented journal MISQE (11 cases), and the handbook of digital innovation by Nambisan et al. (2020) (2 cases). For these outlets, we used the search string ("digital innovation" AND "case") and limited our search to the period till January 31, 2021. Initially, we found 59 cases by following the understanding of digital innovation by Kohli and Melville (2019), Nambisan et al. (2019) and Yoo et al. (2010). After excluding 25 cases that did not provide enough information on the opportunity recognition, our final data set comprised 34 cases. For each case, we identified the involved DT and screened the data to extract why and how the DT influenced opportunity recognition. We then assessed whether the effects of DT on opportunity recognition could be identified and explained (*real-world fidelity*), and whether there were any effects missing (*completeness*). Accordingly, we assigned each case to one or more effects where appropriate (see Appendix 2 for further details on the cases and our coding).

Second, we conducted semi-structured expert interviews (Myers and Newman 2007), which are particular useful for validating what is known but also for gaining new insights (Recker 2013). We selected seven entrepreneurship and innovation scholars in the IS domain and seven practitioners working in digital context, as interviewees, based on a purposive sampling approach (Miles and Huberman 2009) and the following requirements: Each interviewee had to be knowledgeable in our subject of interest and hence needed to have at least 2 years of experience as a researcher or at least 3 years of experience as a practitioner in the fields of innovation and/or digital innovation. In addition, we required innovation

to have published at least one article in our pre-defined set of high-impact journals and/or IS conferences, and practitioners to have led at least one DE or digital innovation project. All interviews were conducted digitally using the video conferencing tools Zoom or Microsoft Teams, and attended and recorded by at least one, mostly two of the co-authors. During the interviews, we presented the – at that time – latest version of the effects of DT on opportunity recognition. After clarifying questions of the interviewee, we discussed the key constructs (i.e., actor, market, resource, opportunity-ideas) and effects on opportunity recognition in general and regarding the four criteria. After each interview, the expert's feedback was reflected in the author-team and resulting changes discussed and potentially integrated (see Appendix 3 for details on the interviews).

Overview of experts for validation in semi-structured interviews

For our validation, we selected seven scholars in the IS domain and seven practitioners working in digital contexts, based on a purposive sampling approach (Miles & Huberman, 2009) and the following requirements: Each interviewee had to be knowledgeable in our subject of interest and hence needed to have at least 2 years of experience as a researcher or at least 3 years of experience as a practitioner in the fields of innovation and/or DE and digital innovation. In addition, we required scholars to have published at least one article in our pre-defined set of high-impact journals and/or IS conferences, and practitioners to have led at least one DE project. All interviews were conducted digitally using the video conferencing tools Zoom or Microsoft Teams, and at-tended and recorded by at least one, mostly two of the co-authors.

Table A9 and A10 provide further information on the experts we interviewed for the validation of our effects, i.e., seven innovation scholars in the IS domain (S1-7) and seven practitioners working in digital context (P1-7). The interviews with these experts were conducted between March 2020 and March 2021. We wanted to ensure that the interviewees could assess our research results from different perspectives. Thereby, as for the innovation scholars, we chose researchers from different academic backgrounds and with differing research foci. This led, for instance, to some interviewees particular interested in the business and management dynamics of DE and other interviewees that study DE from a rather technical perspective. For practitioners working in digital context, we also chose interviewees from different

academic backgrounds, but also from organizations that vary regarding their industry and size to cover a broad variety of organizational types.

Table A9: Innovation and Entrepreneurship Scholars in the IS Domain

ID	Role	Research experience in the IS domain (years)	Academic background	Research focus
S1	Professor	>19	Information Systems	• Business process management<br>• Trust management<br>• Innovation systems
S2	Professor	>12	Economics and Business Administration	• Process management & efficiency<br>• Organizational design<br>• Knowledge management in processes<br>• Effects of digitalization
S3	Post-Doc	>10	Finance, Accounting and Taxation	• Entrepreneurship<br>• Innovation
S4	Ph.D. candidate	> 5	Business Administration	• Value-oriented process management<br>• Agile organizational structures
S5	Ph.D. candidate	>4	Finance and Information Management	• Digital business models<br>• Process innovation<br>• Customer centricity<br>• FinTech and data protection
S6	Ph.D. candidate	>3	Finance and Information Management	• Data-driven, value-oriented process management<br>• Digital innovation & entrepreneurship
S7	Ph.D. candidate	>2	Technology-oriented management	• Innovation management<br>• Digital transformation<br>• IT security

Table A10: Practitioners

ID	Job title	Work experience (years)	Academic background	Industry	Employees	Annual revenue (in €)
P1	IT and DI Project Coordinator	>19	Computer Science	Logistic & Mobility	>16.000	4,702 Billion (2018)
P2	Head of IoT & Asset Management	>6	Business & Information Systems Engineering	Healthcare	> 32.000	6,297 Million (2019/ 2020)
P3	Global Product Manager & Product Owner	>8	Business & Information Systems Engineering	Manufacturing	> 30.000	6.297 Billion (2019/2020)
P4	Consultant	>4	International Management	Business Consulting	>10	N/A (below 200 Million)
P5	Senior Innovation Architect	>3	International Information Systems	Manufacturing	> 20.000	3.5 Billion (2018)
P6	Project Manager Digital Innovation	>3	Applied Research on Engineering Sciences	Manufacturing	>1500	~260 Million (2020)

P7	Business Analyst, Requirements Engineer	>4	Business & Information Systems Engineering	Logistics & Mobility	>36.000	8,946 Million (2019)

Overview of Expert Feedback and Resulting Changes

Table A11 provides an overview of the feedback we gathered during the semi-structured interviews with the innovation and entrepreneurship scholars in the IS domain and practitioners working in digital context, including resulting changes. Chronologically, we started with the interviews with the scholars and received valuable feedback, which we iteratively integrated into the effects. During the first interviews with scholars, we realized that an initial version of the effects was too complex and confusing, which negatively affected its understandability. Thus, we adjusted the framework to be simpler and more intuitive regarding its visualization and readability based on the interviewees' feedback. Overall, we got valuable insights and feedback regarding the effects as well as valuable opportunities for further research which we integrated into the corresponding sections in the paper.

Table A11: Expert Feedback and Resulting Changes

ID	General Feedback	Suggestions for Changes	Resulting Changes (if Applicable) / Comments
**S1**	- Descriptions complementing the effects should clearly define what is in scope and what is out of scope (e.g., in scope are effects of digital technologies and out of scope are contextual factors). Otherwise, the framework would become too confusing	1. Clearly state the scope of the research, e.g., regarding constructs<br>2. Integrate the regulator, e.g., as regulators are fostering but also forcing digital innovation by implementing measures against the COVID-19 pandemic<br>3. Differentiate a microeconomic and macroeconomic perspective, e.g., visualization as a multilayer model with the organization in the core	**1** **Change**: Integration of detailed descriptions to specify the scope of our framework<br>**2** **Change**: Addition of rationale #3.3 that addresses the role of regulators for digital innovation initiation<br>**3** **No change**: Opportunity for further research
**S2**	- The framework lacks visual clarity regarding its focus but also its theoretical foundation<br>- It is unclear, how the digital innovation initiation exactly emerges	4. Present digital technology as a central construct<br>5. Provide initial explanations on how digital technology affects digital innovation initiation	**4** **Change**: Highlighting of digital technology as the central enabler<br>**5** **Change**: Development of an integrated explanation for each effect of digital technology, i.e., rationales
**S3**	- Besides the RBV and MBV, there is the Knowledge-Based view, which is currently not addressed in the visualization of the framework. In addition there are other theoretical perspectives which could fit.	6. Consider a broader set of theoretical perspectives, for instance Service-Dominant Logic and the Knowledge-based view	**6** **No change**: Integration of the MBV and RBV as a result of our literature review but – based on our data set – found little evidence for considering other views
**S4**	- The framework is well structured, but lacks a coherent visualization as there are multiple different types of constructs and relationships	7. Explain the bidirectional arrows and how they affect the constructs<br>8. Remove everything unnecessary from the model and focus on the core, i.e., the effects of digital technology	**7** **Change**: Development of an integrated explanation for each effect of digital technology, i.e., rationales<br>**8** **Change**: Clear focusing of the framework on the effects of digital technology in terms of content and visuals
**S5**	- The framework helps to better understand the impact of digital technology<br>- The effects can be confirmed, considering minor suggestions for wording adjustments	9. Present the arrows clearly to avoid misunderstandings e.g., transitive effects might be misunderstood as two detached arrows instead of one continuous<br>10. Ensure consistent wording of the rationales and clearly state which constructs they are addressing	**9** **Change**: Label of the arrows' edges with consistent numbers for each arrow part.<br>**10** **Change**: Adjustment of the wording of the rationales and implementation of a consistent illustration
**S6**	- The framework can be misunderstood as a process, or structural equation model	11. Adjust the visualisation and labels accordingly	**11** **Change**: Consistent labels (numbers) for arrows' edges
**S7**	- The framework does not address obstacles and barriers caused by effects of digital technologies (e.g., ecosystems specify technical standards and thus limit the freedom of design decisions, creating a hierarchy in which ecosystems specify technical standards)	12. Apply the framework to real-world cases to outline which effects occur together<br>13. Study the difference between B2B and B2C organizations and how this affects the effects of digital technology<br>14. Consider obstacles and limitations of digital technology on digital innovation initiation	**12** **Change**: 34 cases to gain further insights on the effects and to validate our effects<br>**13** **No change**: Opportunity for further research<br>**14** **No change**: Opportunity for further research

**P1**	- The framework gives a good overview by differentiating between direct and indirect effects<br>- The framework's clear distinction of relevant effects of digital technology within the table and explanations (rationales) help to consider and understand them	15. Integrate an economical perspective (e.g., regarding business models)<br>16. Consider that maturity of the digital technology plays a decisive role for successful digital innovation initiation	**15** **No change**: Not in focus, opportunity for further research<br>**16** **No change**: Not in focus, opportunity for further research
**P2**	- The logic and structure of the framework is very clear<br>- The framework helps practitioners to identify opportunities and the rationals provide a first suggestion towards potential next steps to foster digital innovation initiation.	17. Add bidirectional relations within the rationales (e.g., regarding the third effects (#3.1) former partners can become competitors, a scenario could be that Amazon AWS and Azure become competitors because Amazon also provides Web-Services)	**17** **No change:** Not in focus, opportunity for further research
**P3**	- The framework represents a good summary of the interplay between digital technology and digital innovation initiation<br>- The explanations of the effects of digital technology clarify how and why the effects occurs<br>- Other technologies, besides digital technology, can be found in the resource construct	18. Consider interdependencies between the effects, e.g., #2 and #3 might be hard to distinguish in practice as platforms and ecosystems mostly occur together<br>19. Reconsider whether effect #5 and #6 also need an actor as the rationales implicitly include an actor and in practice the generation of an opportunity idea always needs an actor	**18** **No change:** Limitation of our work and opportunity for further research<br>**19** **Change**: Actor explicitly integrated in the rationales and interdependence indicated in the results section
**P4**	- The framework's well-structured and elegant visualization and presentation increases the understandability of the effects of digital technology	20. Consider actor as only construct with relation to DT, because only if DT is used it has influence<br>21. Study digital skills of the actor and how they play a role in opportunity recognition<br>22. Integrate contextual factors and the environment into the framework	**20** **No change:** Data set of articles clearly shows that digital technology influences the resource and market (effect #2 and #3)<br>**21** **No change:** Opportunity for further research<br>**22** **No change:** Opportunity for further research
**P5**	- The framework seem to fit the real-world overall, and all effects are confirmed	23. Consider the supplier perspective and where decisions are made to be able to innovate in a targeted manner	**23** **No change:** Opportunity for further research
**P6**	- In the framework the opportunity-idea is well placed centrally because all arrows end there, and this forms the centre of digital innovation initiation	24. Consider whether there is a direct link between market and resource.<br>25. Consider effect #6 also addressing the actor, as #4 and #6 both do<br>26. Study different industries and different business models due to their different influence on the initiation of digital innovation<br>27. Consider different digital innovation outcome types (e.g., incremental, radical)	**24** **No change:** Opportunity for further research<br>**25** **Change:** see change 19<br>**26** **No change:** Opportunity for further research<br>**27** **No change:** Opportunity for further research
**P7**	- Framework provides a good overview, especially the "From" and "To" perspective is helpful to understand digital innovation initiation within the own organization.	28. Consider bidirectional arrows in the framework to depict digital technology as the result of initiating<br>29. Study the economic perspective, early business cases are essential, preferably in combination with a Minimum Viable Product<br>30. Develop the framework into a digital innovation initiation roadmap for organizations	**28** **No change**: Digital technology is considered a means and an end<br>**29** **No change**: Opportunity for further research<br>**30** **No change**: Opportunity for further research
